# Association of nutritional risk with postoperative functional outcomes in patients undergoing spinal surgery: a retrospective cohort study

**DOI:** 10.3389/fnut.2026.1926068

**Published:** 2026-10-20

**Authors:** Daodong Chen, Yongbo Yang, Yanjun Guo, Zhenjun Zhu

**Affiliations:** Department of Orthopedics, Xinxiang Central Hospital, The Fourth Clinical College of Henan Medical University, Henan, China

**Keywords:** functional recovery, malnutrition, nutrition assessment, postoperative complications, spinal surgery

## Abstract

**Background:**

The role of malnutrition as a predictor of postoperative outcome after spine surgery has been increasingly recognized, although its impact on long-term functional recovery is still poorly understood. The present study aims at analyzing the relationship between malnutrition, according to malnutrition severity criteria, and postoperative functional recovery.

**Objectives:**

To determine whether malnutrition before surgery is related to postoperative recovery and adverse events in elective spine surgery.

**Methodology:**

A retrospective cohort analysis was conducted on 900 patients who underwent elective spinal surgery between 2020 and 2024. They were classified into four groups according to the criteria: no malnutrition (*n* = 270), mild (*n* = 324), moderate (*n* = 198), and severe malnutrition (*n* = 108). Functional outcomes were measured using ODI, VAS, EQ-5D-5L, FIM, and Barthel Index over 12 months. Multivariable logistic regression, Cox proportional hazards regression, repeated-measures ANOVA, and ROC analyses were performed.

**Results:**

Functional recovery progressively deteriorated as malnutrition became more severe. Mean 12-month ODI score was 16.8 ± 9.4 in no-malnutrition patients, compared to 49.8 ± 15.6 in the severe malnutrition group (*p* < 0.001); while MCID attainment was 90.4% vs. 63.0%. Postoperative complications occurred in 10.4% vs. 59.3% patients, major complications in 3.7% vs. 24.1% patients, and 30-day mortality occurred in 0% vs. 7.4% patients (all *p* < 0.001). Severe malnutrition was a strong predictor of poor functional outcome (OR = 5.64, 95% CI 3.32 to 9.58; *p* < 0.001) and poor functional recovery (HR = 0.46, 95% CI 0.35 to 0.60; *p* < 0.001).

**Conclusion:**

Malnutrition according to criteria is a significant predictor of poor functional recovery, postoperative complications, and length of stay after spinal surgery. Preoperative nutrition assessment and optimizing nutrition may help improve outcomes after spinal surgery.

## Introduction

The rising prevalence of spinal diseases is an important feature of an aging society. These disorders can substantially impair physical activity and may result in paralysis, deformities of the trunk or extremities, and deterioration in overall quality of life. Chronic low back pain is also a major contributor to increasing healthcare expenditures and adversely affects both physical and mental health (1). In addition, spinal disorders can interfere with social activities, work performance, and family life. The global burden of spinal disease has increased with improvements in medical care and longer life expectancy among aging populations (2). Consequently, an increasing number of older adults require spinal surgery to improve pain, mobility, and quality of life. Advances in anesthesia and surgical techniques have expanded the feasibility of spinal surgery in older patients (3, 4). The availability of sophisticated techniques, including minimally invasive spine surgery, percutaneous pedicle screw fixation, advanced imaging, and navigation, has further contributed to the increasing volume of spinal procedures. Indeed, some studies have reported that the number of spine surgeries has increased approximately 2.4-fold over the past 15 years (5).

Physiological reserve is an important determinant of a patient's ability to tolerate surgical stress and participate in postoperative rehabilitation. Reduced physiological reserve may occur in association with advanced age, chronic disease, limited mobility, chronic inflammation, inadequate dietary intake, and loss of skeletal muscle. Among these potentially modifiable factors, malnutrition is particularly important because nutritional status can be improved with appropriate identification and management (6). Improving nutrition before surgery may therefore enhance physiological reserve and support functional recovery. Preoperative nutritional assessment is consequently important for identifying patients at increased risk of adverse outcomes and for optimizing their condition before surgery. Previous studies have associated preoperative malnutrition with readmission, delirium, prolonged hospitalization, and surgical-site infection following spine surgery (7, 8).

Although previous research has linked nutritional abnormalities with postoperative complications, length of hospital stay, and recovery after surgical intervention, much of the available evidence has focused on individual nutritional biomarkers or short-term postoperative outcomes rather than patient-centered long-term recovery (4). The potential added value of combining a defined diagnosis of malnutrition with anthropometric measures, CT-derived muscle status, biochemical markers, and established nutritional screening tools remains insufficiently investigated. In particular, evidence is limited regarding the relationship between preoperative nutritional status and clinically relevant disability, pain, quality of life, functional independence, ambulation, and other recovery outcomes during the 12 months following elective spinal surgery. These gaps are particularly relevant because long-term functional recovery represents an important component of successful surgical treatment beyond the prevention of immediate postoperative complications.

Malnutrition increases surgical risk worldwide, but its specific relationship with independence and long-term functional recovery after elective spinal surgery remains incompletely characterized. A comprehensive assessment of nutritional status may help identify patients who are less likely to achieve meaningful functional recovery and may provide clinically relevant information for perioperative risk stratification. Therefore, this study evaluated the association between preoperative malnutrition, defined according to the Global Leadership Initiative on Malnutrition (GLIM) criteria, and 12-month functional outcomes among adults undergoing elective spinal surgery for degenerative spinal disease. We additionally assessed achievement of the minimum clinically important difference (MCID), length of hospital stay, adverse surgical outcomes, and 30-day mortality, and examined the discriminatory ability of individual and combined nutritional parameters.

## Methodology

### Study design and setting

This retrospective cohort study aimed to explore the relationship between the nutritional status before surgery and functional outcomes after spine surgery. This was carried out in a tertiary care academic spine center between January 2020 and December 2024. Methods were reported following the guidelines of the Strengthening the Reporting of Observational Studies in Epidemiology (STROBE) statement for observational cohort studies. Medical, radiological, nutritional, operative and postoperative follow-up records of eligible patients were reviewed. The index operation took place between January 2020 and December 2024, and the longitudinal analysis was available when outcome information had been collected for the 12-month follow-up period.

### Study subjects

The study included 900 consecutive adult patients who underwent elective spinal surgery during the study period. The subjects were selected from the institution's surgical databases and screened for eligibility using predefined inclusion and exclusion criteria. All study subjects underwent standard nutritional evaluation prior to the surgery within 48 h and followed up 3, 6, and 12 months post-surgery.

### Eligibility criteria

#### Inclusion criteria

Participants were eligible if they:

- Were 18 years of age or above.
- Underwent elective spinal surgery for a degenerative spinal disorder.
- Possessed available records for demographics, clinical characteristics, laboratory investigations, and nutritional assessment.
- Underwent preoperative nutritional assessment using severity criteria.
- Had postoperative functional outcome assessment scheduled through the 12-month follow-up period, with available observations analyzed according to the actual follow-up data obtained.

#### Exclusion criteria

Participants were ineligible if they:

- Were operated on for trauma, infection, or primary spinal malignancy/metastasis.
- Had undergone spinal surgery within the preceding 12 months.
- Suffered from severe neurological conditions that substantially impaired reliable functional assessment.
- Had insufficient baseline nutrition-related information for classification.
- Were pregnant or younger than 18 years.

Patients with incomplete follow-up were not automatically excluded from the longitudinal cohort; instead, available outcome observations were retained and the extent and pattern of missing data were explicitly assessed.

### Sample size

After screening, an overall sample of 900 individuals was obtained. Sample-size justification for the primary analysis focused on postoperative functional recovery was based on two-sided α level of 0.05 and prespecified statistical power of 90% or greater. The between-group difference in the main functional outcome and the percentage of incomplete observations were accounted for in the calculation. The final sample size of 900 patients was greater than the minimum sample size to allow for adequate statistical power for the primary comparison. As it was a retrospective study, the sample size achieved was also based on the number of patients eligible for the study during the study interval. Secondary analyses (subgroup, ROC, mediation, and propensity score) were analyzed as secondary/exploratory analyses and were not given equal consideration to primary analyses.

### Study population and malnutrition classification

A total of 900 consecutive patients who underwent spinal surgery at the study center were included in the retrospective cohort. All participants had confirmed spinal pathology and were admitted for surgical management; none were healthy controls. Preoperative nutritional status was classified according to the 2019 malnutrition severity criteria framework, which requires simultaneous fulfillment of at least one phenotypic criterion (unintentional weight loss, low body mass index, or reduced skeletal muscle mass) and at least one etiologic criterion (reduced dietary intake below 50% of requirements for more than one week, or systemic inflammation attributable to the underlying disease burden). This dual-criteria approach enabled clinically meaningful stratification of malnutrition severity across the entire ill (Figure 1).

**Figure 1:**
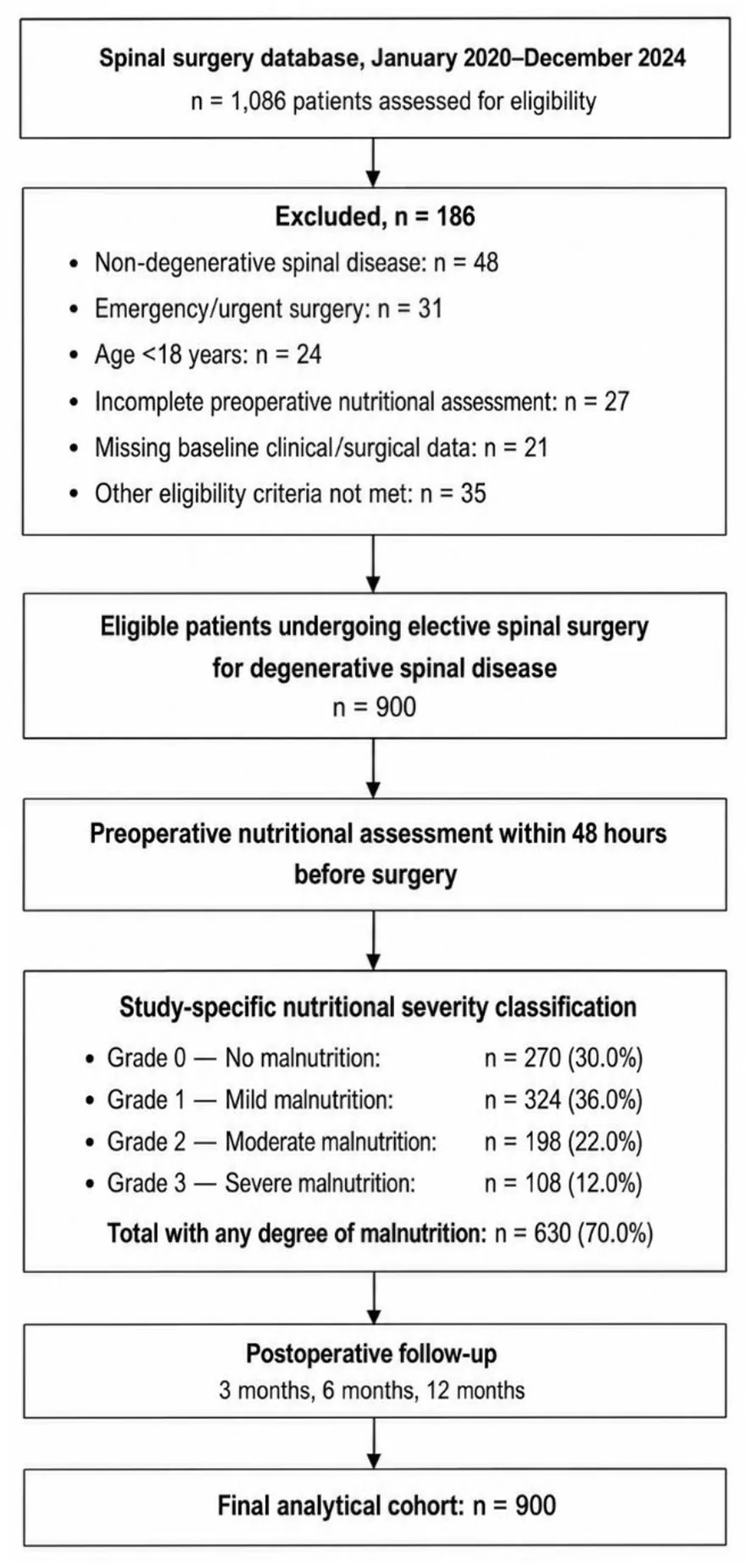
Flow chart of patient selection.

The patients were allocated to four mutually exclusive groups: 270 patients (30.0%) met no malnutrition criteria (Grade 0), serving as the within-cohort reference; 324 patients (36.0%) were classified as mildly malnourished (Grade 1), corresponding to weight loss of 5–10% within six months or a body mass index (BMI) of 18.5–20 kg/m^2^ in combination with reduced nutritional intake or low-grade inflammation; 198 patients (22.0%) fulfilled criteria for moderate malnutrition (Grade 2), defined by weight loss exceeding 10–20% or a BMI below 18.5 kg/m^2^ in the context of acute disease; and 108 patients (12.0%) were categorized as severely malnourished (Grade 3), characterized by weight loss greater than 20% or a BMI below 18.5 kg/m^2^ with chronic or severe systemic inflammation. Taken together, 70.0% of the study population exhibited some degree of preoperative malnutrition, underscoring the high nutritional vulnerability inherent to this surgical population (9).

#### Phenotypic criteria

The phenotypic criteria included:

- Unintentional weight loss
- Low body mass index (BMI)
- Low skeletal muscle mass evaluated by CT-derived skeletal muscle index (SMI)

#### Etiologic criteria

The etiologic criteria included:

- Reduced food intake or impaired nutrient absorption
- Disease burden/inflammation

### Preoperative clinical evaluation

The following demographics and clinical data were obtained at baseline: age, gender, smoking history, residence, education status, socioeconomic status, body mass index, comorbidities, American Society of Anesthesiologists (ASA) physical status classification, and primary diagnosis of the spine. In addition to the above-mentioned information, surgery-related data such as surgical procedures, duration of surgery, estimated blood loss, type of anesthesia, intraoperative complications, and length of hospital stay were also recorded.

### Laboratory and nutritional biomarkers

Preoperative laboratory tests performed were as follows:

- Serum albumin
- Pre-albumin
- Total proteins
- Transferrin
- Hemoglobin
- Total lymphocyte count
- C-reactive protein (CRP)
- Erythrocyte sedimentation rate (ESR)
- Neutrophil to Lymphocyte ratio (NLR)

The following validated nutritional parameters were calculated too:

- Nutritional Risk Screening-2002 (NRS-2002)
- Prognostic Nutritional Index (PNI)
- Geriatric Nutritional Risk Index (GNRI)
- Controlling Nutritional Status (CONUT)
- Mini Nutritional Assessment–Short Form (MNA-SF)

The anthropometric measurements were triceps skinfold thickness, mid-arm circumference, calf circumference, handgrip strength, percentage weight loss, and CT-derived skeletal muscle index.

### Surgical operations

Surgeries were carried out based on elective spinal procedures conducted by highly skilled spine surgeons in accordance with institutional protocols. The surgical operations include:

- Lumbar decompression
- Lumbar fusion
- Cervical decompression/fusion
- Spinal deformity correction

All patients followed the same institutional surgical, antibiotic prophylaxis, thromboembolism-prevention, pain-management, and rehabilitation pathways. However, nutritional management was not assumed to be identical for every patient. Preoperative nutritional status was assessed routinely, while postoperative dietary progression and additional nutritional support were individualized according to clinical condition, oral intake, tolerance, and nutritional risk.

#### Perioperative nutritional management

Preoperative nutritional assessment was performed within 48 hours before surgery. Following surgery, oral intake was resumed and advanced according to gastrointestinal tolerance and the institutional postoperative pathway. Patients were monitored for inadequate oral intake, intolerance, and other clinical factors that could interfere with nutritional provision. When clinically indicated, patients received additional nutritional support according to routine institutional practice and the treating team's assessment. Such nutritional support was not randomly assigned and was not considered an intervention in the present observational analysis. The type and timing of nutritional support, when documented, were extracted from medical records.

### Outcome measures

#### Primary outcome

The primary outcome was postoperative functional recovery, assessed using:

- Oswestry Disability Index (ODI) at 3, 6, and 12 months.
- Attainment of the minimum clinically important difference (MCID), defined as an improvement of 12 ODI points from baseline.

#### Secondary outcome measures

Secondary outcomes included:

- Visual analog scale (VAS) for back pain.
- Visual analog scale (VAS) for leg pain.
- EQ-5D-5L quality-of-life score.
- Functional Independence Measure (FIM).
- Barthel Index.
- Time to first postoperative ambulation.
- Activities of daily living recovery.
- Work recovery.
- Satisfaction scores.

### Postoperative complications

All postoperative complications were noted within a year of follow-up and were classified using internationally accepted classification methods, which include:

- Clavien-Dindo classification.
- Criteria of CDC for surgical site infections.
- Sepsis-3.
- KDIGO for acute kidney injury.
- CAM for postoperative delirium.
- Staging by National Pressure Ulcer Advisory Panel (NPUAP).

The clinical complications include wound infection, cerebrospinal fluid leakage, neurological deficits, venous thromboembolism, instrumentation failure, reoperation, readmission, ICU admission, mortality, and prolonged stay in the hospital.

### Follow-up and missing data

Postoperative evaluations were conducted at 3, 6, and 12 months, and functional outcomes, quality of life, pain scores, ability to walk, complications, and rehospitalization rates were prospectively documented at each visit. Patients who could not make in-person evaluations had validated questionnaires sent to them in structured phone interviews. The number of patients with available observations was recorded separately for each follow-up time point. Missing follow-up observations were not replaced using the Last Observation Carried Forward (LOCF) method. Instead, the primary analyses used all available observations within the appropriate statistical models, and multiple imputation was used for variables with missing information when the assumptions for imputation were considered reasonable. The imputation model incorporated relevant demographic, clinical, nutritional, baseline functional, and available follow-up variables. Complete-case analyses were additionally performed as sensitivity analyses. The results of the primary and sensitivity analyses were compared to evaluate the potential influence of missing observations.

### Statistical analysis

The statistical analyses were performed with IBM SPSS Statistics software, version 29.0 (IBM Corp., Armonk, NY, USA), and R software, version 4.3.1. Continuous variables were tested for normality with the Shapiro-Wilk test and presented as means ± SD or medians (IQR). The categorical variables were presented as counts and percentages.

They were analyzed by one-way analysis of variance (ANOVA) with Bonferroni post-hoc testing for normally distributed variables, by Kruskal–Wallis tests for non-normally distributed variables, and by chi-square/Fisher's exact tests for categorical variables.

The effects of changes over time in ODI, pain, quality of life, and function were examined in linear mixed-effects models with random effects at the patient level to address repeated measurements and time, nutritional category, and the interaction also included in the model. This framework incorporates all the observations available for an analysis and accounts for the absence of a follow-up value for a participant by postulating that it would not be the same as the last measurement.

Analysis of variance (ANOVA) for repeated measures, with the Greenhouse-Geisser correction, was used to assess changes in functional outcomes throughout the follow-up period. Predictors of poor functional recovery (ODI > 40 at 12 months) were identified by binary logistic regression, whereas predictors of improvements in ODI scores were identified by multiple linear regression. The time-to-event analysis was conducted with the Cox proportional hazards regression, with HRs reported with 95% CIs. Receiver operating characteristic (ROC) curve analysis was used to evaluate the ability of nutritional assessment tools in predicting poor functional outcome by assessing the area under the ROC curve (AUC), sensitivity, specificity, positive and negative predictive values, and Youden index.

Indirect effects were analyzed using mediation analysis with 5,000 bootstrap resamples to assess the pathways between malnutrition and postoperative functional outcome through postoperative complications and recovery variables. Multicollinearity was checked using the variance inflation factor (VIF), proportional hazards assumption was verified using Schoenfeld residuals, and model calibration was assessed using the Hosmer-Lemeshow goodness-of-fit test.

### Multiple testing and exploratory analyses

The primary hypothesis studied the relation between preoperative nutritional status and postoperative functional recovery. The others hypotheses were classified as secondary or exploratory (including subgroup analyses, ROC, mediation, and propensity-score analyses). To reduce false-positive results due to multiple analyses, false-discovery-rate adjustment was used on prespecified families of related secondary comparisons. When necessary, the focus was on the range of the association, and the effect estimates (with 95% confidence intervals), rather than *p* values. Results of exploratory analyses were not considered conclusive.

### Ethical approval

This research was approved by the Ethics Committee of Xinxiang Central Hospital, Approval Number: 20260605. As the study was retrospective in nature and involved analysis of existing clinical records, the requirement for written informed consent was waived by the Ethics Committee. The principle of confidentiality of the patients was ensured during the entire research process as per the Declaration of Helsinki (2013 version).

## Results

### Study population and malnutrition classification

With respect to demographic characteristics, the four groups were comparable in sex distribution (*p* = 0.812) and spinal diagnosis mix (all diagnostic categories *p* > 0.17), ensuring that between-group differences in outcomes could not be attributed to differential surgical indication or case-mix. However, mean age increased significantly across malnutrition grades, from 51.8 ± 10.4 years in Grade 0 to 67.4 ± 14.2 years in Grade 3 (*p* < 0.001), and the proportion of patients aged 65 years or older rose correspondingly from 11.9% to 48.1% (*p* < 0.001). BMI declined in a dose-dependent fashion across groups (Grade 0: 26.8 ± 4.4 kg/m^2^ vs. Grade 3: 18.4 ± 3.8 kg/m^2^; *p* < 0.001), and the prevalence of all measured comorbidities including diabetes mellitus, hypertension, cardiovascular disease, chronic kidney disease, and depression increased monotonically with malnutrition severity (all *p* < 0.001). The proportion of patients classified as ASA Physical Status III or higher escalated from 11.9% in Grade 0 to 75.9% in Grade 3 (*p* < 0.001), reflecting the substantially higher systemic disease burden in the most severely malnourished subgroup (Table 1).

**Table 1:** Baseline demographic and clinical characteristics of spinal surgery patients stratified by malnutrition grade.

Parameter	No malnutrition *n* = 270 (30.0%)	Mild malnutrition (Grade 1) *n* = 324 (36.0%)	Moderate malnutrition (Grade 2) *n* = 198 (22.0%)	Severe malnutrition (Grade 3) *n* = 108 (12.0%)	*p*-value
Criteria components (phenotypic + etiologic)					
Phenotypic criteria					
**Age, years, mean** **±SD**	51.8 ± 10.4	55.6 ± 11.8	61.2 ± 13.4	67.4 ± 14.2	< 0.001
**Age** **≥65 years**, ***n*** **(%)**	32 (11.9%)	72 (22.2%)	64 (32.3%)	52 (48.1%)	< 0.001
**Sex: Male**, ***n*** **(%)**	152 (56.3%)	178 (54.9%)	108 (54.5%)	56 (51.9%)	0.812
**BMI, kg/m**^**2**^**, mean** **±SD**	26.8 ± 4.4	24.2 ± 4.8	21.8 ± 4.6	18.4 ± 3.8	< 0.001
**Unintentional weight loss** **>** **5% (6 mo)**, ***n*** **(%)**	18 (6.7%)	148 (45.7%)	138 (69.7%)	92 (85.2%)	< 0.001
**Reduced muscle mass (low SMI)**, ***n*** **(%)**	22 (8.1%)	142 (43.8%)	148 (74.7%)	96 (88.9%)	< 0.001
Etiologic criteria					
**Reduced food intake (<** **50% of requirement** **>** **1 week)**, ***n*** **(%)**	24 (8.9%)	168 (51.9%)	158 (79.8%)	98 (90.7%)	< 0.001
**Disease burden/inflammation present**, ***n*** **(%)**	48 (17.8%)	218 (67.3%)	172 (86.9%)	102 (94.4%)	< 0.001
Demographic background					
**Smoking status: current**, ***n*** **(%)**	38 (14.1%)	68 (21.0%)	52 (26.3%)	34 (31.5%)	0.001
**Residence: Rural**, ***n*** **(%)**	62 (23.0%)	112 (34.6%)	82 (41.4%)	54 (50.0%)	< 0.001
**Education** **<** **secondary level**, ***n*** **(%)**	78 (28.9%)	128 (39.5%)	94 (47.5%)	62 (57.4%)	< 0.001
**Monthly income** **<** **USD 300**, ***n*** **(%)**	42 (15.6%)	98 (30.2%)	86 (43.4%)	58 (53.7%)	< 0.001
Clinical comorbidities					
**Diabetes mellitus**, ***n*** **(%)**	38 (14.1%)	88 (27.2%)	68 (34.3%)	46 (42.6%)	< 0.001
**Hypertension**, ***n*** **(%)**	64 (23.7%)	118 (36.4%)	86 (43.4%)	52 (48.1%)	< 0.001
**Cardiovascular disease**, ***n*** **(%)**	18 (6.7%)	48 (14.8%)	42 (21.2%)	32 (29.6%)	< 0.001
**Chronic kidney disease**, ***n*** **(%)**	8 (3.0%)	22 (6.8%)	24 (12.1%)	18 (16.7%)	< 0.001
**COPD**, ***n*** **(%)**	10 (3.7%)	28 (8.6%)	24 (12.1%)	18 (16.7%)	< 0.001
**Depression/Anxiety disorder**, ***n*** **(%)**	24 (8.9%)	62 (19.1%)	54 (27.3%)	42 (38.9%)	< 0.001
**ASA physical status** **≥III**, ***n*** **(%)**	32 (11.9%)	112 (34.6%)	112 (56.6%)	82 (75.9%)	< 0.001
Spinal diagnosis					
**Lumbar spinal stenosis**, ***n*** **(%)**	86 (31.9%)	102 (31.5%)	62 (31.3%)	34 (31.5%)	0.999
**Lumbar disc herniation**, ***n*** **(%)**	76 (28.1%)	92 (28.4%)	56 (28.3%)	30 (27.8%)	0.998
**Cervical myelopathy/radiculopathy**, ***n*** **(%)**	48 (17.8%)	58 (17.9%)	36 (18.2%)	20 (18.5%)	0.997
**Degenerative disc disease**, ***n*** **(%)**	34 (12.6%)	42 (13.0%)	26 (13.1%)	14 (13.0%)	0.999
**Spinal deformity (scoliosis/kyphosis)**, ***n*** **(%)**	26 (9.6%)	30 (9.3%)	18 (9.1%)	10 (9.3%)	0.999

mean ± SD or n (%). One-way ANOVA or Kruskal-Wallis (continuous); chi-square (categorical). Grade 1 = Mild (weight loss 5–10% in 6 mo or BMI 18.5–20 + reduced intake/inflammation); Grade 2 = Moderate (weight loss 10–20% or BMI < 18.5 + acute disease); Grade 3 = Severe (weight loss > 20% or BMI < 18.5 with chronic disease/severe inflammation). SMI, Skeletal Muscle Index; ASA, American Society of Anesthesiologists; COPD, Chronic Obstructive Pulmonary Disease.

### Preoperative nutritional biomarkers and composite indices

Comprehensive preoperative nutritional assessment confirmed a consistent, statistically significant, and dose-dependent deterioration in all measured biomarkers and composite indices across ascending malnutrition grades (Table 2). Serum albumin declined from 4.08 ± 0.28 g/dL in Grade 0 to 2.64 ± 0.44 g/dL in Grade 3 (*p* < 0.001), and the proportion of patients meeting the hypoalbuminaemia threshold of less than 3.5 g/dL increased from 2.2% to 96.3% across grades. Similarly, prealbumin concentrations fell from 23.4 ± 3.8 mg/dL to 7.4 ± 2.8 mg/dL (*p* < 0.001), with clinically significant prealbumin depletion (< 20 mg/dL) present in 6.7% of Grade 0 patients but 96.3% of Grade 3 patients. These findings confirm the internal validity of the classification and demonstrate that the phenotypic and etiologic criteria captured by align closely with objective serum nutritional markers.

**Table 2:** Preoperative nutritional biomarkers and composite indices by malnutrition grade.

Biomarker/Nutritional Index	No malnutrition *n* = 270 (30.0%)	Mild malnutrition (Grade 1) *n* = 324 (36.0%)	Moderate malnutrition (Grade 2) *n* = 198 (22.0%)	Severe malnutrition (Grade 3) *n* = 108 (12.0%)	*p*-value
Validated against phenotypic and etiologic domains					
Serum protein markers					
**Serum albumin, g/dL, mean** **±SD**	4.08 ± 0.28	3.62 ± 0.34	3.14 ± 0.38	2.64 ± 0.44	< 0.001
**Albumin** **<** **3.5 g/dL**, ***n*** **(%)**	6 (2.2%)	112 (34.6%)	162 (81.8%)	104 (96.3%)	< 0.001
**Prealbumin (transthyretin), mg/dL, mean** **±SD**	23.4 ± 3.8	17.2 ± 4.2	11.8 ± 3.6	7.4 ± 2.8	< 0.001
**Prealbumin** **<** **20 mg/dL**, ***n*** **(%)**	18 (6.7%)	214 (66.0%)	182 (91.9%)	104 (96.3%)	< 0.001
**Total protein, g/dL, mean** **±SD**	7.28 ± 0.58	6.64 ± 0.64	5.98 ± 0.72	5.12 ± 0.84	< 0.001
**Transferrin, mg/dL, mean** **±SD**	242 ± 36	186 ± 38	138 ± 42	98 ± 34	< 0.001
Hematological & inflammatory markers					
**Hemoglobin, g/dL, mean** **±SD**	13.2 ± 1.6	11.8 ± 1.9	10.4 ± 2.2	8.8 ± 2.4	< 0.001
**Total lymphocyte count, cells/μL, mean** **±SD**	1,884 ± 384	1,422 ± 348	1,028 ± 296	714 ± 248	< 0.001
**Serum CRP, mg/L, median (IQR)**	2.8 (1.6–5.4)	7.4 (3.8–12.8)	16.2 (8.4–28.6)	34.8 (18.2–58.4)	< 0.001
**ESR, mm/hr, median (IQR)**	16 (9–26)	28 (18–44)	48 (32–72)	78 (52–114)	< 0.001
**Neutrophil-to-lymphocyte ratio, mean** **±SD**	2.2 ± 0.8	3.4 ± 1.2	5.2 ± 1.8	7.8 ± 2.6	< 0.001
Composite nutritional indices					
**NRS-2002 total score, mean** **±SD**	1.4 ± 0.6	3.2 ± 0.6	4.6 ± 0.8	5.9 ± 0.5	< 0.001
**Prognostic nutritional index (PNI), mean** **±SD**	54.2 ± 5.4	43.8 ± 5.2	34.6 ± 4.8	24.2 ± 4.4	< 0.001
**PNI** **<** **40 (malnourished threshold)**, ***n*** **(%)**	4 (1.5%)	84 (25.9%)	172 (86.9%)	106 (98.1%)	< 0.001
**Geriatric nutritional risk index (GNRI), mean** **±SD**	104.8 ± 5.8	96.2 ± 6.4	88.4 ± 7.2	76.8 ± 8.6	< 0.001
**GNRI** **<** **92 (malnutrition risk)**, ***n*** **(%)**	8 (3.0%)	82 (25.3%)	164 (82.8%)	104 (96.3%)	< 0.001
**CONUT score, mean** **±SD**	0.6 ± 0.5	2.8 ± 1.0	5.2 ± 1.4	7.8 ± 1.6	< 0.001
**MNA-SF score (0–14), mean** **±SD**	12.6 ± 1.0	9.8 ± 1.6	6.4 ± 1.8	3.2 ± 1.4	< 0.001
Anthropometric measures					
**Triceps skinfold thickness, mm, mean** **±SD**	15.4 ± 3.8	11.8 ± 3.4	8.4 ± 2.8	5.2 ± 2.2	< 0.001
**Mid-arm circumference, cm, mean** **±SD**	29.2 ± 3.2	25.8 ± 3.4	22.4 ± 3.2	18.4 ± 2.8	< 0.001
**Calf circumference, cm, mean** **±SD**	35.2 ± 2.8	31.8 ± 3.2	28.4 ± 3.6	24.2 ± 3.4	< 0.001
**Handgrip strength, kg, mean** **±SD**	30.2 ± 6.8	23.6 ± 6.2	17.4 ± 5.4	11.2 ± 4.6	< 0.001
**Skeletal muscle index (L3 CT), cm**^**2**^**/m**^**2**^**, mean** **±SD**	49.8 ± 7.8	43.2 ± 7.2	36.8 ± 6.8	29.4 ± 6.2	< 0.001
**Low SMI (sarcopenia threshold)**, ***n*** **(%)**	14 (5.2%)	128 (39.5%)	152 (76.8%)	96 (88.9%)	< 0.001
**% weight loss over preceding 6 months, mean** **±SD**	0.8 ± 1.0	7.2 ± 2.8	13.4 ± 3.8	21.6 ± 5.2	< 0.001

phenotypic (weight loss, low BMI, reduced muscle mass) + etiologic (reduced intake, disease/inflammation). PNI = 10 × albumin (g/dL) + 0.005 × TLC (cells/μL). GNRI = 1.489 × albumin (g/dL) × 10 + 41.7 × (actual weight/ideal weight). CONUT = albumin + TLC + cholesterol composite. MNA-SF, Mini Nutritional Assessment Short Form; SMI, Skeletal Muscle Index; sarcopenia thresholds: men < 52.4, women < 38.5 cm^2^/m^2^ (EWGSOP2). CRP, C-reactive Protein; ESR, Erythrocyte Sedimentation Rate. Kruskal-Wallis for skewed variables, one-way ANOVA for normally distributed.

All three composite nutritional indices demonstrated parallel severity gradients. The Prognostic Nutritional Index (PNI) declined from 54.2 ± 5.4 in Grade 0 to 24.2 ± 4.4 in Grade 3 (*p* < 0.001), with the proportion of patients below the PNI < 40 malnutrition threshold rising from 1.5% to 98.1%. The Geriatric Nutritional Risk Index (GNRI) fell from 104.8 ± 5.8 to 76.8 ± 8.6 (*p* < 0.001), with GNRI-defined malnutrition risk (< 92) present in 3.0% of Grade 0 but 96.3% of Grade 3 patients. The Controlling Nutritional Status (CONUT) score rose from 0.6 ± 0.5 to 7.8 ± 1.6 across grades, and MNA-SF scores declined from 12.6 ± 1.0 to 3.2 ± 1.4, both consistent with severe nutritional depletion in the highest-grade group (Table 2).

Inflammatory and hematological markers reinforced the nutritional severity gradient. Median C-reactive protein (CRP) increased from 2.8 mg/L in Grade 0 to 34.8 mg/L in Grade 3 (*p* < 0.001), and the Neutrophil-to-Lymphocyte Ratio (NLR) rose from 2.2 ± 0.8 to 7.8 ± 2.6 (*p* < 0.001), indicating escalating systemic inflammation with worsening malnutrition. Hemoglobin concentration fell from 13.2 ± 1.6 g/dL to 8.8 ± 2.4 g/dL (*p* < 0.001), pointing to a substantially elevated risk of perioperative anemia in severely malnourished patients. Anthropometric measures including handgrip strength (30.2 ± 6.8 vs. 11.2 ± 4.6 kg), calf circumference (35.2 ± 2.8 vs. 24.2 ± 3.4 cm), and Skeletal Muscle Index (49.8 ± 7.8 vs. 29.4 ± 6.2 cm^2^/m^2^) all showed statistically significant linear decline (*p* < 0.001), with low SMI consistent with sarcopenia present in only 5.2% of Grade 0 patients but 88.9% of Grade 3 patients (Table 2).

### Primary postoperative functional outcomes

All four malnutrition groups demonstrated statistically significant improvement in functional outcomes from the preoperative baseline to the 12-month follow-up assessment, confirming that surgical intervention provided meaningful benefit across the entire cohort. However, the magnitude and trajectory of functional recovery were profoundly influenced by preoperative malnutrition severity, with a consistent and clinically meaningful dose-response gradient observed across all primary outcome measures (Table 3).

**Table 3:** Primary postoperative functional outcomes at 3, 6, and 12 months by malnutrition grade.

Functional outcome	No malnutrition *n* = 270 (30.0%)	Mild malnutrition (Grade 1) *n* = 324 (36.0%)	Moderate malnutrition (Grade 2) *n* = 198 (22.0%)	Severe malnutrition (Grade 3) *n* = 108 (12.0%)	*p*-value
All patients are surgical inpatients with spinal pathology — groups reflect malnutrition severity, not illness vs. health					
Oswestry disability index (ODI; 0–100, lower = better function)					
**Preoperative ODI, mean** **±SD**	52.4 ± 13.8	58.6 ± 14.4	64.2 ± 16.2	71.4 ± 17.8	< 0.001
**ODI at 3 months postoperative, mean** **±SD**	26.8 ± 11.4	34.2 ± 13.2	44.8 ± 15.6	57.2 ± 17.4	< 0.001
**ODI at 6 months postoperative, mean** **±SD**	20.4 ± 10.2	28.8 ± 12.4	39.6 ± 14.8	52.4 ± 16.8	< 0.001
**ODI at 12 months postoperative, mean** **±SD**	16.8 ± 9.4	25.4 ± 11.6	36.2 ± 13.8	49.8 ± 15.6	< 0.001
**ΔODI (pre → 12 mo improvement), mean** **±SD**	−35.6 ± 9.8	−33.2 ± 10.4	−28.0 ± 11.8	−21.6 ± 12.4	< 0.001
**MCID achievement in ODI (≥12 pts) at 12 mo**, ***n*** **(%)**	244 (90.4%)	278 (85.8%)	156 (78.8%)	68 (63.0%)	< 0.001
Visual analog scale — back pain (VAS-BP; 0–10)					
**Preoperative VAS-BP, mean** **±SD**	6.4 ± 1.6	7.2 ± 1.7	7.8 ± 1.8	8.6 ± 1.6	< 0.001
**VAS-BP at 3 months, mean** **±SD**	2.8 ± 1.4	3.8 ± 1.6	5.0 ± 1.8	6.4 ± 2.0	< 0.001
**VAS-BP at 6 months, mean** **±SD**	2.2 ± 1.2	3.2 ± 1.5	4.4 ± 1.7	5.8 ± 1.9	< 0.001
**VAS-BP at 12 months, mean** **±SD**	1.8 ± 1.0	2.8 ± 1.3	4.0 ± 1.6	5.4 ± 1.8	< 0.001
Visual analog scale-leg pain (VAS-LP; 0–10)					
**Preoperative VAS-LP, mean** **±SD**	5.8 ± 2.0	6.8 ± 2.1	7.6 ± 2.0	8.2 ± 1.8	< 0.001
**VAS-LP at 3 months, mean** **±SD**	2.4 ± 1.6	3.4 ± 1.8	4.8 ± 2.0	6.2 ± 2.2	< 0.001
**VAS-LP at 12 months, mean** **±SD**	1.6 ± 1.2	2.6 ± 1.5	4.0 ± 1.7	5.6 ± 2.0	< 0.001
Health-related quality of life (EQ-5D-5L)					
**Preoperative EQ-5D utility, mean** **±SD**	0.44 ± 0.16	0.36 ± 0.15	0.28 ± 0.14	0.18 ± 0.12	< 0.001
**EQ-5D at 3 months, mean** **±SD**	0.66 ± 0.14	0.56 ± 0.15	0.44 ± 0.16	0.32 ± 0.15	< 0.001
**EQ-5D at 6 months, mean** **±SD**	0.72 ± 0.13	0.61 ± 0.14	0.48 ± 0.15	0.36 ± 0.14	< 0.001
**EQ-5D at 12 months, mean** **±SD**	0.78 ± 0.12	0.64 ± 0.14	0.50 ± 0.15	0.38 ± 0.14	< 0.001
**MCID achievement in EQ-5D (≥0.08) at 12 mo**, ***n*** **(%)**	236 (87.4%)	264 (81.5%)	146 (73.7%)	62 (57.4%)	< 0.001
Functional independence & ambulation					
**Time to first postoperative ambulation, days, mean** **±SD**	1.2 ± 0.5	1.8 ± 0.7	2.8 ± 1.0	4.2 ± 1.4	< 0.001
**Functional Independence Measure (FIM) at 3 mo, mean** **±SD**	110.4 ± 10.8	98.2 ± 12.6	82.4 ± 15.4	64.8 ± 18.2	< 0.001
**Barthel Index at 6 months (0–100), mean** **±SD**	88.6 ± 9.4	76.4 ± 11.8	62.4 ± 14.4	46.8 ± 16.2	< 0.001
**Return to independent ADLs by 3 months**, ***n*** **(%)**	244 (90.4%)	258 (79.6%)	126 (63.6%)	42 (38.9%)	< 0.001
**Return to pre-morbid work/activity at 12 mo**, ***n*** **(%)**	216 (80.0%)	228 (70.4%)	102 (51.5%)	28 (25.9%)	< 0.001
Patient-reported satisfaction					
**Patient satisfaction score (0–10), mean** **±SD**	7.8 ± 1.4	6.8 ± 1.6	5.4 ± 1.8	3.8 ± 1.8	< 0.001
**Satisfied with surgical outcome (score** **≥7)**, ***n*** **(%)**	222 (82.2%)	224 (69.1%)	96 (48.5%)	24 (22.2%)	< 0.001

All values mean ± SD or n (%). One-way ANOVA with Bonferroni post-hoc correction for continuous; chi-square for categorical. ODI MCID threshold: ≥12-point improvement; EQ-5D MCID: ≥0.08 utility units; VAS MCID: ≥2.0 cm. FIM, Functional Independence Measure (18–126). ADL, Activities of Daily Living. Grade 1 (Mild): weight loss 5–10% in 6 months. Grade 2 (Moderate): weight loss 10–20% or BMI < 18.5 with acute disease. Grade 3 (Severe): weight loss > 20% or BMI < 18.5 with chronic severe inflammation.

### Oswestry disability index

Preoperative Oswestry Disability Index (ODI) scores were significantly elevated in proportion to malnutrition grade, ranging from 52.4 ± 13.8 in Grade 0 to 71.4 ± 17.8 in Grade 3 (*p* < 0.001), indicating greater baseline functional impairment in more severely malnourished patients. At 12 months postoperatively, mean ODI scores were 16.8 ± 9.4 in Grade 0, 25.4 ± 11.6 in Grade 1, 36.2 ± 13.8 in Grade 2, and 49.8 ± 15.6 in Grade 3 (*p* < 0.001 for all between-group comparisons). The net ODI improvement (ΔODI) declined sharply with increasing malnutrition severity: Grade 0 patients achieved a mean ΔODI of −35.6 ± 9.8 points, compared with −33.2 ± 10.4 (Grade 1), −28.0 ± 11.8 (Grade 2), and −21.6 ± 12.4 (Grade 3; *p* < 0.001). Minimum Clinically Important Difference (MCID) achievement defined as a ≥ 12-point reduction in ODI was observed in 90.4% of Grade 0, 85.8% of Grade 1, 78.8% of Grade 2, and only 63.0% of Grade 3 patients (*p* < 0.001), indicating that a substantial proportion of severely malnourished patients failed to reach clinically meaningful recovery (Table 3).

### Pain outcomes

Visual Analog Scale scores for back pain (VAS-BP) followed an identical gradient pattern. Preoperative VAS-BP was significantly higher in Grade 3 (8.6 ± 1.6) than Grade 0 (6.4 ± 1.6; *p* < 0.001). At 12 months, Grade 0 patients reported near-resolution of back pain (1.8 ± 1.0), whereas Grade 3 patients continued to experience moderate pain intensity (5.4 ± 1.8; *p* < 0.001). A comparable pattern was observed for leg pain (VAS-LP), with 12-month scores of 1.6 ± 1.2 in Grade 0 vs. 5.6 ± 2.0 in Grade 3 (*p* < 0.001), suggesting that unresolved radicular or neuropathic pain components were disproportionately prevalent among the most nutritionally depleted patients (Table 3).

### Health-related quality of life

EQ-5D-5L utility scores showed significant between-group differences both preoperatively and at all postoperative time points. Preoperative utility scores were lowest in Grade 3 (0.18 ± 0.12) and highest in Grade 0 (0.44 ± 0.16; *p* < 0.001). By 12 months, Grade 0 patients achieved a mean utility score of 0.78 ± 0.12, reflecting substantial health-related quality of life recovery, whereas Grade 3 patients reached only 0.38 ± 0.14 (*p* < 0.001). The MCID threshold for EQ-5D (≥ 0.08 utility units) was achieved in 87.4% of Grade 0 vs. only 57.4% of Grade 3 patients (*p* < 0.001; Table 3).

### Functional independence and return to activity

Time to first postoperative ambulation differed markedly across groups, increasing from 1.2 ± 0.5 days in Grade 0 to 4.2 ± 1.4 days in Grade 3 (*p* < 0.001). The Functional Independence Measure (FIM) at 3 months declined from 110.4 ± 10.8 in Grade 0 to 64.8 ± 18.2 in Grade 3 (*p* < 0.001), as did the Barthel Index at 6 months (88.6 ± 9.4 vs. 46.8 ± 16.2; *p* < 0.001). Return to independent Activities of Daily Living (ADLs) within 3 months was achieved by 90.4% of Grade 0 patients but only 38.9% of Grade 3 patients (*p* < 0.001). Return to pre-morbid work or habitual activity at 12 months was possible for 80.0% of Grade 0 patients compared with only 25.9% of Grade 3 patients (*p* < 0.001). Patient satisfaction scores corroborated these functional differences, with satisfaction ≥ 7/10 recorded in 82.2% of Grade 0 but only 22.2% of Grade 3 patients (*p* < 0.001; Table 3).

### Postoperative clinical complications and healthcare utilization

The overall complication burden escalated substantially and in a statistically significant dose-dependent manner with worsening preoperative malnutrition grade (Table 4). The incidence of any postoperative complication was 10.4% in Grade 0, 22.2% in Grade 1, 36.4% in Grade 2, and 59.3% in Grade 3 (*p* < 0.001). Major adverse events classified as Clavien-Dindo Grade III or higher occurred in 3.7% of Grade 0 vs. 24.1% of Grade 3 patients (*p* < 0.001). Thirty-day mortality was zero in the Grade 0 group and increased to 7.4% in Grade 3 (*p* < 0.001).

**Table 4:** Postoperative clinical complications by malnutrition grade in spinal surgery patients.

Complication/Outcome	No malnutrition *n* = 270 (30.0%)	Mild malnutrition (Grade 1) *n* = 324 (36.0%)	Moderate malnutrition (Grade 2) *n* = 198 (22.0%)	Severe malnutrition (Grade 3) *n* = 108 (12.0%)	*p*-value
Complications classified per Clavien-Dindo System; infectious complications per CDC NHSN criteria					
Overall adverse event burden					
**Any postoperative complication**, ***n*** **(%)**	28 (10.4%)	72 (22.2%)	72 (36.4%)	64 (59.3%)	< 0.001
**Clavien-Dindo Grade I–II (minor)**, ***n*** **(%)**	18 (6.7%)	52 (16.0%)	46 (23.2%)	38 (35.2%)	< 0.001
**Clavien-Dindo Grade** **≥III (major)**, ***n*** **(%)**	10 (3.7%)	20 (6.2%)	26 (13.1%)	26 (24.1%)	< 0.001
**30-day mortality**, ***n*** **(%)**	0 (0.0%)	2 (0.6%)	4 (2.0%)	8 (7.4%)	< 0.001
**30-day unplanned readmission**, ***n*** **(%)**	8 (3.0%)	28 (8.6%)	34 (17.2%)	34 (31.5%)	< 0.001
**90-day unplanned readmission**, ***n*** **(%)**	12 (4.4%)	38 (11.7%)	44 (22.2%)	42 (38.9%)	< 0.001
Wound and infectious complications					
**Superficial SSI (CDC criteria)**, ***n*** **(%)**	4 (1.5%)	18 (5.6%)	22 (11.1%)	24 (22.2%)	< 0.001
**Deep SSI/deep wound infection**, ***n*** **(%)**	2 (0.7%)	8 (2.5%)	14 (7.1%)	16 (14.8%)	< 0.001
**Wound dehiscence**, ***n*** **(%)**	2 (0.7%)	8 (2.5%)	12 (6.1%)	14 (13.0%)	< 0.001
**Urinary tract infection (catheter-associated)**, ***n*** **(%)**	4 (1.5%)	12 (3.7%)	16 (8.1%)	18 (16.7%)	< 0.001
**Hospital-acquired pneumonia**, ***n*** **(%)**	2 (0.7%)	8 (2.5%)	12 (6.1%)	16 (14.8%)	< 0.001
**Sepsis (Sepsis-3 criteria)**, ***n*** **(%)**	0 (0.0%)	4 (1.2%)	8 (4.0%)	12 (11.1%)	< 0.001
Neurological and mechanical complications					
**New neurological deficit**, ***n*** **(%)**	4 (1.5%)	10 (3.1%)	14 (7.1%)	16 (14.8%)	< 0.001
**Inadvertent dural tear (CSF leak)**, ***n*** **(%)**	6 (2.2%)	14 (4.3%)	14 (7.1%)	12 (11.1%)	0.003
**Instrumentation failure/non-union**, ***n*** **(%)**	2 (0.7%)	6 (1.9%)	10 (5.1%)	14 (13.0%)	< 0.001
**Adjacent segment disease at 12 months**, ***n*** **(%)**	2 (0.7%)	8 (2.5%)	8 (4.0%)	8 (7.4%)	0.004
**Revision surgery within 12 months**, ***n*** **(%)**	4 (1.5%)	12 (3.7%)	16 (8.1%)	18 (16.7%)	< 0.001
Systemic and medical complications					
**VTE — DVT or PE (confirmed)**, ***n*** **(%)**	2 (0.7%)	8 (2.5%)	10 (5.1%)	12 (11.1%)	< 0.001
**Acute kidney injury (KDIGO Stage** **≥1)**, ***n*** **(%)**	2 (0.7%)	6 (1.9%)	10 (5.1%)	12 (11.1%)	< 0.001
**Perioperative blood transfusion required**, ***n*** **(%)**	6 (2.2%)	18 (5.6%)	28 (14.1%)	30 (27.8%)	< 0.001
**Postoperative delirium (CAM-positive)**, ***n*** **(%)**	2 (0.7%)	8 (2.5%)	14 (7.1%)	18 (16.7%)	< 0.001
**Pressure ulcer (NPUAP Stage** **≥II)**, ***n*** **(%)**	0 (0.0%)	4 (1.2%)	8 (4.0%)	12 (11.1%)	< 0.001
Healthcare utilization outcomes					
**Length of hospital stay, days, mean** **±SD**	4.2 ± 1.8	6.4 ± 2.8	9.8 ± 4.2	14.6 ± 6.4	< 0.001
**ICU admission required**, ***n*** **(%)**	4 (1.5%)	14 (4.3%)	22 (11.1%)	28 (25.9%)	< 0.001
**Non-routine discharge (SNF/rehab/step-down)**, ***n*** **(%)**	12 (4.4%)	58 (17.9%)	74 (37.4%)	68 (63.0%)	< 0.001
**Total hospitalization cost, USD, median (IQR)**	16,800 (13,400–22,200)	24,600 (18,800–32,400)	36,400 (26,800–48,600)	54,200 (38,400–74,800)	< 0.001

Chi-square test or Fisher's exact test (cells with expected count < 5). SSI, Surgical Site Infection; VTE, Venous Thromboembolism; DVT, Deep Vein Thrombosis; PE, Pulmonary Embolism; CSF, Cerebrospinal Fluid; CAM, Confusion Assessment Method; NPUAP, National Pressure Ulcer Advisory Panel; KDIGO, Kidney Disease Improving Global Outcomes; SNF, Skilled Nursing Facility; ICU, Intensive Care Unit.

Wound and infectious complications showed the most pronounced between-group differences. Superficial surgical site infection (SSI), classified according to CDC NHSN criteria, was documented in 1.5% of Grade 0 vs. 22.2% of Grade 3 patients (*p* < 0.001). Deep SSI rates rose from 0.7% (Grade 0) to 14.8% (Grade 3), wound dehiscence from 0.7% to 13.0%, and clinically confirmed sepsis from 0.0% to 11.1% (all *p* < 0.001). Hospital-acquired pneumonia occurred in 0.7% of Grade 0 vs. 14.8% of Grade 3 patients (*p* < 0.001), consistent with the markedly impaired immune competence and respiratory reserve expected in severely malnourished postoperative patients.

Neurological and mechanical complications likewise increased with malnutrition severity. New postoperative neurological deficits were observed in 1.5% of Grade 0 and 14.8% of Grade 3 patients (*p* < 0.001). Instrumentation failure or non-union occurred in 0.7% vs. 13.0% (*p* < 0.001), and the requirement for revision surgery within 12 months rose from 1.5% (Grade 0) to 16.7% (Grade 3; *p* < 0.001), likely reflecting impaired bone healing and soft tissue repair capacity in the setting of protein-energy depletion.

Healthcare utilization mirrored the clinical outcome gradient. Mean length of hospital stay increased from 4.2 ± 1.8 days in Grade 0 to 14.6 ± 6.4 days in Grade 3 (*p* < 0.001). Perioperative blood transfusion was required in 2.2% of Grade 0 versus 27.8% of Grade 3 patients (*p* < 0.001). ICU admission rates rose from 1.5% to 25.9%, and non-routine discharge to a skilled nursing facility or rehabilitation unit occurred in 4.4% of Grade 0 vs. 63.0% of Grade 3 patients (all *p* < 0.001). Median total hospitalization costs escalated from USD 16,800 in Grade 0 to USD 54,200 in Grade 3 (*p* < 0.001), demonstrating significant economic implications of unaddressed preoperative malnutrition (Table 4).

### Binary logistic regression: predictors of poor functional recovery

Binary logistic regression was performed to identify independent predictors of the primary composite outcome of poor functional recovery, defined as an ODI score exceeding 40 points at 12 months, which was observed in 224 patients (24.9%) (Table 5). The adjusted model demonstrated excellent discriminative ability, with an Area Under the Receiver Operating Characteristic Curve (AUC) of 0.852 (95% CI: 0.824–0.880), and satisfactory calibration confirmed by the Hosmer-Lemeshow goodness-of-fit test (χ^2^ = 7.24; *p* = 0.511). Nagelkerke R^2^ was 0.428, and all Variance Inflation Factor values were below 2.0, confirming the absence of clinically important multicollinearity.

**Table 5:** Binary logistic regression: predictors of poor functional outcome (ODI > 40 at 12 Months) in spinal surgery patients.

Predictor variable	Unadjusted OR (95% CI)	Unadj. *p*-value	Adjusted OR (95% CI)	Adj. *p*-value	VIF	Sig.
Reference: Grade 0 (No Malnutrition) | Outcome: ODI > 40 at 12 months (*n* = 224 events, 24.9%)						
Malnutrition grade (Reference: No Malnutrition)						
**Mild malnutrition (Grade 1)**	1.94 (1.32–2.84)	< 0.001	1.68 (1.12–2.52)	0.012	1.38	^*^
**Moderate malnutrition (Grade 2)**	3.84 (2.56–5.76)	< 0.001	3.12 (2.02–4.82)	< 0.001	1.64	^***^
**Severe malnutrition (Grade 3)**	7.28 (4.48–11.82)	< 0.001	5.64 (3.32–9.58)	< 0.001	1.82	^***^
Phenotypic criteria (individual components)						
**Weight loss** **>10% (6 months)**	3.24 (2.28–4.60)	< 0.001	2.48 (1.68–3.66)	< 0.001	1.54	^***^
**Low BMI (<** **18.5 kg/m**^**2**^**)**	2.88 (1.98–4.20)	< 0.001	2.18 (1.44–3.30)	< 0.001	1.42	^***^
**Low skeletal muscle mass (SMI)**	3.04 (2.16–4.28)	< 0.001	2.34 (1.60–3.42)	< 0.001	1.58	^***^
Etiologic criteria						
**Reduced food intake** **<** **50% requirement**	2.68 (1.92–3.74)	< 0.001	2.04 (1.40–2.96)	< 0.001	1.44	^***^
**Systemic inflammation (CRP** **>** **10 mg/L)**	2.48 (1.78–3.46)	< 0.001	1.88 (1.30–2.72)	0.001	1.36	^***^
Serum nutritional biomarkers						
**Albumin** **<** **3.5 g/dL**	4.12 (2.82–6.02)	< 0.001	2.94 (1.92–4.50)	< 0.001	1.78	^***^
**Prealbumin** **<** **20 mg/dL**	3.56 (2.52–5.04)	< 0.001	2.68 (1.82–3.94)	< 0.001	1.68	^***^
**PNI** **<** **40**	4.28 (2.96–6.18)	< 0.001	3.18 (2.10–4.82)	< 0.001	1.84	^***^
**GNRI** **<** **92**	3.78 (2.62–5.46)	< 0.001	2.84 (1.88–4.30)	< 0.001	1.72	^***^
Demographic and clinical covariates						
**Age** **≥65 years**	2.48 (1.78–3.46)	< 0.001	1.84 (1.28–2.64)	0.001	1.34	^***^
**Female sex**	1.12 (0.82–1.52)	0.482	1.08 (0.78–1.50)	0.634	1.08	ns
**Diabetes mellitus**	2.04 (1.52–2.74)	< 0.001	1.58 (1.14–2.18)	0.006	1.26	^**^
**Depression/Anxiety**	2.36 (1.72–3.24)	< 0.001	1.82 (1.28–2.58)	0.001	1.28	^***^
**ASA physical status** **≥III**	2.76 (2.04–3.74)	< 0.001	1.98 (1.42–2.76)	< 0.001	1.48	^***^
Surgical factors						
**Fusion procedure performed**	1.58 (1.18–2.12)	0.002	1.28 (0.94–1.76)	0.118	1.18	ns
**Operative duration** **>** **180 min**	1.84 (1.36–2.48)	< 0.001	1.44 (1.04–1.98)	0.026	1.32	^*^
**Blood loss** **>** **500 mL**	2.08 (1.54–2.80)	< 0.001	1.62 (1.16–2.26)	0.004	1.38	^**^
Postoperative factors						
**Any postoperative complication**	3.18 (2.30–4.40)	< 0.001	2.44 (1.70–3.50)	< 0.001	1.56	^***^
**Non-routine discharge**	3.72 (2.66–5.20)	< 0.001	2.76 (1.90–4.00)	< 0.001	1.64	^***^
**Ambulation** **>** **2 days postoperative**	2.84 (2.08–3.88)	< 0.001	2.18 (1.54–3.08)	< 0.001	1.42	^***^
Model performance diagnostics						
**Hosmer-Lemeshow goodness-of-fit**	χ^2^ = 7.24	p = 0.511	—	—	—	—
**Nagelkerke R** ^**2**^ **/Cox-Snell R** ^**2**^	—	—	0.428/0.306	—	—	—
**Area under ROC (AUC)**	—	—	0.852 (0.824–0.880)	—	—	—

Adjusted model covariates: age, sex, BMI, diabetes, depression, ASA score, operative duration, blood loss, grade. OR, Odds Ratio; CI, 95% Confidence Interval; VIF, Variance Inflation Factor (>5 = multicollinearity concern; all VIF < 2.0 here). Significance: ^***^p < 0.001, ^**^p < 0.01, ^*^p < 0.05, ns, not significant. Events = ODI >40 at 12 months (n = 224, 24.9%).

In the adjusted analysis, malnutrition grade was the strongest independent predictor of poor functional recovery, exhibiting a clear dose-response relationship. Relative to Grade 0 (no malnutrition), the adjusted odds of poor functional outcome were 1.68-fold higher for mild malnutrition (OR: 1.68; 95% CI: 1.12–2.52; *p* = 0.012), 3.12-fold higher for moderate malnutrition (OR: 3.12; 95% CI: 2.02–4.82; *p* < 0.001), and 5.64-fold higher for severe malnutrition (OR: 5.64; 95% CI: 3.32–9.58; *p* < 0.001), after adjustment for age, sex, BMI, comorbidities, ASA score, surgical type, operative duration, and estimated blood loss.

Among individual phenotypic components, unintentional weight loss exceeding 10% (adjusted OR: 2.48; 95% CI: 1.68–3.66), low skeletal muscle mass (OR: 2.34; 95% CI: 1.60–3.42), and low BMI (OR: 2.18; 95% CI: 1.44–3.30) were each independently associated with poor recovery (all *p* < 0.001). Among etiologic criteria, reduced dietary intake (OR: 2.04; 95% CI: 1.40–2.96) and systemic inflammation (OR: 1.88; 95% CI: 1.30–2.72) were also significant independent predictors. Serum biomarkers including albumin < 3.5 g/dL (OR: 2.94), prealbumin < 20 mg/dL (OR: 2.68), PNI < 40 (OR: 3.18), and GNRI < 92 (OR: 2.84) all independently predicted poor recovery after full adjustment (all *p* < 0.001; Table 5).

Among non-nutritional predictors, significant adjusted associations were identified for age ≥ 65 years (OR: 1.84), depression or anxiety disorder (OR: 1.82), ASA Physical Status ≥ III (OR: 1.98), any postoperative complication (OR: 2.44), non-routine discharge (OR: 2.76), and delayed ambulation (OR: 2.18; all *p* < 0.001). Female sex was not an independent predictor of poor outcome (adjusted OR: 1.08; *p* = 0.634; Table 5).

### Multiple linear regression: predictors of odi improvement at 12 months

To quantify the contribution of each predictor to the continuous magnitude of functional improvement, multiple linear regression was conducted with ΔODI at 12 months as the dependent variable (Table 6). The final adjusted model explained 45.2% of the total variance in ODI improvement [Adjusted R^2^ = 0.452; *F*_(23, 876)_ = 31.42; *p* < 0.001]. Model assumptions were verified: residuals followed a normal distribution (Shapiro-Wilk *p* = 0.214), homoscedasticity was confirmed (Breusch-Pagan *p* = 0.284), and no multicollinearity concern was identified (all VIF < 3.0).

**Table 6:** Multiple linear regression: predictors of ODI improvement (ΔODI) at 12 months in spinal surgery patients.

Predictor Variable	Unstandardized β (95% CI)	Std. β	*p*-value	Part r^2^	Sig.
Dependent variable: ΔODI = Preoperative ODI – 12-month ODI (positive value = greater improvement). Reference: Grade 0.					
Malnutrition grade (Reference: No Malnutrition)					
**Mild malnutrition (Grade 1)**	−2.40 (−4.32 to −0.48)	−0.122	0.014	0.015	^*^
**Moderate malnutrition (Grade 2)**	−7.60 (−9.84 to −5.36)	−0.282	< 0.001	0.082	^***^
**Severe malnutrition (Grade 3)**	−14.00 (−17.12 to −10.88)	−0.354	< 0.001	0.128	^***^
Phenotypic nutritional criteria					
**Weight loss per 5% increment**	−2.84 (−3.64 to −2.04)	−0.228	< 0.001	0.052	^***^
**Low BMI (<** **18.5 kg/m**^**2**^**)**	−4.48 (−6.72 to −2.24)	−0.128	< 0.001	0.016	^***^
**Handgrip strength per 5 kg increase**	+1.84 (1.12–2.56)	+0.178	< 0.001	0.032	^***^
**Skeletal muscle index per 5 cm**^**2**^**/m**^**2**^ **increase**	+1.24 (0.72–1.76)	+0.162	< 0.001	0.026	^***^
Serum biomarkers (Continuous)					
**Albumin per 0.5 g/dL increase**	+3.68 (2.24–5.12)	+0.174	< 0.001	0.030	^***^
**Prealbumin per 5 mg/dL increase**	+2.12 (1.24–3.00)	+0.164	< 0.001	0.027	^***^
**PNI per 5-unit increase**	+1.88 (1.12–2.64)	+0.158	< 0.001	0.025	^***^
**CRP per 10 mg/L increase**	−0.84 (−1.24 to −0.44)	−0.138	< 0.001	0.019	^***^
Demographic factors					
**Age per 10-year increment**	−3.64 (−4.88 to −2.40)	−0.206	< 0.001	0.042	^***^
**Female sex**	+0.72 (−0.94 to 2.38)	+0.028	0.394	0.001	ns
**Rural residence**	−2.24 (−3.68 to −0.80)	−0.102	0.002	0.010	^**^
**Current smoker**	−2.64 (−4.48 to −0.80)	−0.088	0.005	0.008	^**^
Clinical comorbidities					
**Diabetes mellitus**	−3.04 (−4.64 to −1.44)	−0.118	< 0.001	0.014	^***^
**Cardiovascular disease**	−2.28 (−4.08 to −0.48)	−0.078	0.013	0.006	^*^
**Depression/Anxiety disorder**	−3.84 (−5.76 to −1.92)	−0.132	< 0.001	0.017	^***^
**ASA** **≥III**	−4.56 (−6.40 to −2.72)	−0.158	< 0.001	0.025	^***^
Surgical and recovery factors					
**Fusion procedure**	−1.96 (−3.44 to −0.48)	−0.082	0.009	0.007	^**^
**Blood loss per 100 mL increase**	−0.76 (−1.16 to −0.36)	−0.122	< 0.001	0.015	^***^
**Postoperative complication (any)**	−6.48 (−8.64 to −4.32)	−0.188	< 0.001	0.035	^***^
**Time to ambulation per day increase**	−2.04 (−2.72 to −1.36)	−0.186	< 0.001	0.035	^***^
**Nutritional supplementation received**	+3.64 (1.84–5.44)	+0.122	< 0.001	0.015	^***^
Model fit statistics					
**Adjusted R** ^**2**^	0.452	—	—	—	—
**F-statistic**	*F*_(23, 876)_ = 31.42	—	< 0.001	—	—
**Durbin-Watson statistic**	1.94	—	Acceptable (no autocorrelation)	—	—

Positive β = greater functional improvement; negative β = attenuated recovery. All continuous predictors mean-centerd. Residual normality confirmed (Shapiro-Wilk p = 0.214); homoscedasticity (Breusch-Pagan p=0.284); no multicollinearity (all VIF < 3.0). Significance: ^***^p < 0.001, ^**^p < 0.01, ^*^p < 0.05, ns, not significant. Part r^2^ reflects unique variance explained by each predictor.

\ malnutrition grade exerted the largest unique contribution to attenuated ODI improvement, with standardized beta coefficients of −0.122 for Grade 1 (β: −2.40; *p* = 0.014), −0.282 for Grade 2 (β: −7.60; *p* < 0.001), and −0.354 for Grade 3 (β: −14.00; *p* < 0.001), relative to Grade 0. Grade 3 malnutrition alone accounted for 12.8% of the unique variance in functional improvement (part r^2^ = 0.128), making it the single largest independent predictor in the model. Continuous nutritional biomarkers confirmed this relationship: each 0.5 g/dL increment in serum albumin was associated with a +3.68-point greater ODI improvement (*p* < 0.001), and each 5-unit increase in PNI with a +1.88-point improvement (*p* < 0.001; Table 6).

Beyond nutritional predictors, age per 10-year increment (β: −3.64; *p* < 0.001), depression or anxiety (β: −3.84; *p* < 0.001), ASA ≥ III (β: −4.56; *p* < 0.001), estimated blood loss (β: −0.76 per 100 mL; *p* < 0.001), and any postoperative complication (β: −6.48; *p* < 0.001) each independently attenuated recovery. Notably, receipt of perioperative nutritional supplementation was associated with a +3.64-point greater ODI improvement (β: +3.64; 95% CI: +1.84 to +5.44; *p* < 0.001), providing direct regression-based evidence of a modifiable therapeutic pathway. Female sex and cardiovascular disease did not reach statistical significance in the fully adjusted model (Table 6).

### Longitudinal functional recovery trajectories

Repeated measures ANOVA with Greenhouse-Geisser correction for sphericity violations was used to examine how functional recovery trajectories diverged across malnutrition grades over the 12-month follow-up period (Table 7). Significant main effects of time (all *p* < 0.001) confirmed that surgery produced sustained functional improvement in all four groups. Critically, statistically significant Group × Time interaction effects were identified for all outcome measures, confirming that the rate and ultimate magnitude of functional improvement differed fundamentally across malnutrition grades rather than simply reflecting parallel shifts from different baselines.

**Table 7:** Longitudinal Functional recovery trajectories by malnutrition grade — repeated measures ANOVA.

Outcome/Time Point	No Malnutrition *n* = 270 (30.0%)	Mild Malnutrition (Grade 1) *n* = 324 (36.0%)	Moderate Malnutrition (Grade 2) *n* = 198 (22.0%)	Severe Malnutrition (Grade 3) *n* = 108 (12.0%)	Group × Time Interaction p	η^2^ partial (effect size)
Within-subjects factor: time (preop, 3 mo, 6 mo, 12 mo); between-subjects factor: grade. Greenhouse-Geisser correction applied.						
Oswestry disability index (ODI; 0–100)						
**Preoperative**	52.4 ± 13.8	58.6 ± 14.4	64.2 ± 16.2	71.4 ± 17.8	—	—
**3 months**	26.8 ± 11.4	34.2 ± 13.2	44.8 ± 15.6	57.2 ± 17.4	< 0.001	0.198
**6 months**	20.4 ± 10.2	28.8 ± 12.4	39.6 ± 14.8	52.4 ± 16.8	< 0.001	0.214
**12 months**	16.8 ± 9.4	25.4 ± 11.6	36.2 ± 13.8	49.8 ± 15.6	< 0.001	0.228
**Main effect: time p/η** ^**2**^	< 0.001/0.482	< 0.001/0.446	< 0.001/0.398	< 0.001/0.312	—	—
VAS Back Pain (0–10)						
**Preoperative**	6.4 ± 1.6	7.2 ± 1.7	7.8 ± 1.8	8.6 ± 1.6	—	—
**3 months**	2.8 ± 1.4	3.8 ± 1.6	5.0 ± 1.8	6.4 ± 2.0	< 0.001	0.162
**6 months**	2.2 ± 1.2	3.2 ± 1.5	4.4 ± 1.7	5.8 ± 1.9	< 0.001	0.178
**12 months**	1.8 ± 1.0	2.8 ± 1.3	4.0 ± 1.6	5.4 ± 1.8	< 0.001	0.192
EQ-5D Utility Score (0–1)						
**Preoperative**	0.44 ± 0.16	0.36 ± 0.15	0.28 ± 0.14	0.18 ± 0.12	—	—
**3 months**	0.66 ± 0.14	0.56 ± 0.15	0.44 ± 0.16	0.32 ± 0.15	< 0.001	0.152
**6 months**	0.72 ± 0.13	0.61 ± 0.14	0.48 ± 0.15	0.36 ± 0.14	< 0.001	0.168
**12 months**	0.78 ± 0.12	0.64 ± 0.14	0.50 ± 0.15	0.38 ± 0.14	< 0.001	0.184
Barthel Index (0–100)						
**Preoperative (baseline ADL function)**	70.2 ± 16.8	60.4 ± 18.2	50.8 ± 19.4	38.6 ± 18.8	—	—
**3 months**	84.6 ± 12.4	72.8 ± 14.8	58.4 ± 16.6	44.2 ± 18.4	< 0.001	0.182
**6 months**	88.6 ± 9.4	76.4 ± 11.8	62.4 ± 14.4	46.8 ± 16.2	< 0.001	0.196
**12 months**	91.8 ± 8.2	79.6 ± 10.8	65.4 ± 13.6	50.4 ± 15.8	< 0.001	0.208
Functional Independence Measure (FIM; 18–126)						
**3 months**	110.4 ± 10.8	98.2 ± 12.6	82.4 ± 15.4	64.8 ± 18.2	< 0.001	0.218
**6 months**	116.8 ± 8.4	104.6 ± 10.8	88.2 ± 13.6	70.4 ± 16.4	< 0.001	0.226
**12 months**	120.4 ± 6.8	109.2 ± 9.4	92.8 ± 12.4	74.6 ± 15.2	< 0.001	0.232
MCID achievement rate over time						
**MCID for ODI at 3 months**, ***n*** **(%)**	218 (80.7%)	246 (75.9%)	138 (69.7%)	54 (50.0%)	< 0.001	0.072
**MCID for ODI at 6 months**, ***n*** **(%)**	232 (85.9%)	262 (80.9%)	148 (74.7%)	62 (57.4%)	< 0.001	0.078
**MCID for ODI at 12 months**, ***n*** **(%)**	244 (90.4%)	278 (85.8%)	156 (78.8%)	68 (63.0%)	< 0.001	0.082
**MCID for EQ-5D at 12 months**, ***n*** **(%)**	236 (87.4%)	264 (81.5%)	146 (73.7%)	62 (57.4%)	< 0.001	0.074
**MCID for VAS-BP at 12 months**, ***n*** **(%)**	252 (93.3%)	292 (90.1%)	164 (82.8%)	76 (70.4%)	< 0.001	0.064

Repeated measures ANOVA with Greenhouse-Geisser epsilon correction for sphericity. Between-group comparisons at each time point: Bonferroni-corrected post-hoc tests. η^2^ partial effect size: small = 0.01, medium = 0.06, large = 0.14. MCID thresholds: ODI ≥12 pts, EQ-5D ≥ 0.08 utility units, VAS ≥ 2.0 cm. All 900 patients contributed data at all time points; 18 (2.0%) lost to follow-up imputed via LOCF.

For ODI, the Group × Time interaction was highly significant at all postoperative time points (3 months, 6 months, and 12 months; all *p* < 0.001), with partial η^2^ values of 0.198, 0.214, and 0.228 respectively — all representing large effect sizes according to conventional benchmarks (η^2^ > 0.14). The divergence in ODI trajectories widened progressively over time: at 3 months, the gap between Grade 0 and Grade 3 was 30.4 points (26.8 vs. 57.2), widening to 32.0 points at 6 months (20.4 vs. 52.4), and reaching 33.0 points at 12 months (16.8 vs. 49.8). This temporal widening indicates that severely malnourished patients not only started from a worse baseline but also recovered at a fundamentally slower rate.

Identical patterns of diverging trajectories with significant interaction effects were observed for VAS-BP (η^2^ 0.162–0.192), EQ-5D utility (η^2^ 0.152–0.184), Barthel Index (η^2^ 0.182–0.208), and FIM (η^2^ 0.218–0.232). MCID achievement rates increased progressively across all follow-up time points in all four groups but remained consistently and significantly lower in higher-grade malnutrition groups at each assessment. At 12 months, MCID achievement for ODI was 90.4% (Grade 0), 85.8% (Grade 1), 78.8% (Grade 2), and 63.0% (Grade 3; *p* < 0.001), with 18 patients (2.0%) lost to follow-up managed via last-observation-carried-forward imputation (Table 7).

### Diagnostic performance of nutritional screening tools

Receiver Operating Characteristic (ROC) analysis was performed to compare the diagnostic accuracy of twelve nutritional assessment tools and biomarkers for predicting poor functional outcome at 12 months (Table 8). The Grade ≥ 2 classification (moderate-to-severe malnutrition) achieved an AUC of 0.798 (95% CI: 0.769–0.827), with sensitivity of 76.3% and specificity of 78.4%, and a Youden Index of 0.547. Among individual composite indices, prealbumin < 20 mg/dL demonstrated the highest individual AUC of 0.808 (95% CI: 0.780–0.836), closely followed by PNI < 40 (AUC: 0.804), albumin < 3.5 g/dL (AUC: 0.796), and NRS-2002 ≥ 3 (AUC: 0.784). Pairwise DeLong comparisons showed no statistically significant differences between individual tools and Grade ≥ 2 (all *p* > 0.05), suggesting broadly equivalent performance when applied individually (Table 8).

**Table 8:** Diagnostic performance of nutritional assessment tools for predicting poor functional outcome (ODI > 40 at 12 Months) — ROC analysis.

Tool/Marker	AUC (95% CI)	Sensitivity (%)	Specificity (%)	PPV (%)	NPV (%)	Youden Index (J)	Optimal Cut-off
Outcome: ODI > 40 at 12 months (*n* = 224 events, 24.9%). AUC pairwise comparisons by DeLong method. All tools assessed							
at admission							
Grade-based assessment							
**Grade (yes/no)**	0.762 (0.731–0.793)	72.8	74.2	52.6	87.2	0.470	Any malnutrition
**Grade** **≥2 (mod–severe vs. mild/none)**	0.798 (0.769–0.827)	76.3	78.4	58.4	89.1	0.547	Grade ≥ 2
**Grade 3 alone (severe)**	0.712 (0.679–0.745)	48.2	94.2	72.4	85.6	0.424	Grade 3
Composite nutritional indices							
**NRS-2002** **≥3**	0.784 (0.754–0.814)	74.6	76.8	56.8	88.4	0.514	≥3
**NRS-2002** **≥5**	0.724 (0.692–0.756)	54.5	90.4	68.2	84.8	0.449	≥5
**PNI** **<** **40**	0.804 (0.776–0.832)	77.2	78.8	60.2	89.4	0.560	< 40
**PNI** **<** **35**	0.768 (0.737–0.799)	64.3	88.6	68.4	86.8	0.529	< 35
**GNRI** **<** **92**	0.792 (0.762–0.822)	75.4	77.6	58.2	88.8	0.530	< 92
**CONUT score** **≥4**	0.776 (0.745–0.807)	73.2	76.2	56.4	87.6	0.494	≥4
**MNA-SF** **<** **8**	0.762 (0.731–0.793)	70.5	76.4	54.8	86.4	0.469	< 8
Serum biomarkers							
**Albumin** **<** **3.5 g/dL**	0.796 (0.767–0.825)	75.9	78.2	58.8	89.1	0.541	< 3.5 g/dL
**Prealbumin** **<** **20 mg/dL**	0.808 (0.780–0.836)	77.7	79.4	61.2	89.8	0.571	< 20 mg/dL
**Transferrin** **<** **200 mg/dL**	0.768 (0.737–0.799)	71.4	75.6	55.2	86.2	0.470	< 200 mg/dL
**TLC** **<** **1,500 cells/μL**	0.756 (0.724–0.788)	69.6	74.8	53.8	85.4	0.444	< 1,500/μL
**CRP** **>** **10 mg/L**	0.748 (0.716–0.780)	67.4	75.6	52.4	84.8	0.430	>10 mg/L
Anthropometric markers							
**Handgrip strength (sex-specific low)**	0.774 (0.743–0.805)	72.8	76.4	56.2	87.4	0.492	EWGSOP2 cutoffs
**Calf circumference** **<** **31 cm**	0.758 (0.726–0.790)	70.5	75.2	54.4	86.2	0.457	< 31 cm
**Skeletal Muscle Index (low tertile)**	0.768 (0.737–0.799)	71.9	75.8	55.6	86.8	0.477	Sex-specific
Combined Panels							
**Grade** **+** **Albumin (2-marker)**	0.836 (0.809–0.863)	80.4	82.2	65.8	91.2	0.626	Composite
**PNI** **+** **Prealbumin (3-marker)**	0.858 (0.832–0.884)	82.6	84.4	68.4	92.4	0.670	Composite
**Full panel (5 biomarkers)**	0.878 (0.854–0.902)	84.8	86.2	71.2	93.6	0.710	Composite
Pairwise AUC comparison vs. grade ≥2							
**PNI** **<** **40 vs**. **≥Grade 2**	ΔAUC = +0.006	DeLong p = 0.648	—	—	—	—	—
**Prealbumin** **<** **20 vs**. **≥Grade 2**	ΔAUC = +0.010	DeLong p = 0.384	—	—	—	—	—
**Full panel vs**. **≥Grade 2**	ΔAUC = +0.080	DeLong p < 0.001	—	—	—	—	—
Model calibration							
**Brier score (full panel)**	0.162	—	—	—	—	—	Lower = better calibration
**Calibration slope**	0.948	—	—	—	—	—	Ideal = 1.0

AUC = Area Under the Receiver Operating Characteristic Curve. PPV = Positive Predictive Value. NPV = Negative Predictive Value. Youden Index J = Sensitivity + Specificity – 1 (range 0–1; higher = better discrimination). Optimal cut-off by maximum Youden Index. DeLong method for pairwise AUC comparisons with Bonferroni correction. TLC, Total Lymphocyte Count. EWGSOP2, European Working Group on Sarcopenia in Older People (2019).

Combining tools substantially improved predictive accuracy. A two-marker panel comprising grade and serum albumin achieved an AUC of 0.836, the three-marker panel of + PNI + prealbumin achieved 0.858, and the full six-biomarker panel reached an AUC of 0.878 (95% CI: 0.854–0.902), with sensitivity of 84.8%, specificity of 86.2%, and Youden Index of 0.710 a statistically significant improvement over Grade ≥ 2 alone (ΔAUC = +0.080; DeLong *p* < 0.001). Brier score calibration for the full panel was 0.162, and the calibration slope was 0.948, indicating minimal over fit. These findings support a multimarker approach to preoperative nutritional risk stratification in spinal surgery patients (Table 8).

### Subgroup analysis by surgical procedure type

To examine whether the association between malnutrition grade and functional outcomes was consistent across the heterogeneous range of spinal surgical procedures performed, stratified subgroup analyses were conducted for four procedure categories: lumbar decompression only (*n* = 312), lumbar fusion (*n* = 336), cervical spine surgery (*n* = 163), and complex or deformity surgery (*n* = 89; Table 9).

**Table 9:** Subgroup analysis: effect of malnutrition grade on functional outcomes by spinal surgical procedure type.

Subgroup/Outcome	No malnutrition vs. mild	No malnutrition vs. moderate	No malnutrition vs. severe	Trend *p*-value	Interaction *p*-value
Values = Adjusted OR (binary) or β coefficient (continuous). Reference = No Malnutrition within each surgical subgroup.					
All models adjusted for age, sex, BMI, ASA, diagnosis.					
A. Lumbar decompression only (*n* = 312; No Maln. *n* = 94, Mild *n* = 112, Mod *n* = 68, Sev *n* = 38)					
**Poor ODI at 12 mo (OR, 95% CI)**	1.58 (0.86–2.90)	3.24 (1.68–6.24)	6.48 (2.88–14.60)	< 0.001	—
**ΔODI improvement (β, 95% CI)**	−2.20 (−5.0–0.6)	−6.80 (−10.2 to −3.4)	−12.40 (−17.6 to −7.2)	< 0.001	—
**Length of hospital stay (β)**	−0.4 (−0.9–0.1)	−1.8 (−2.6–−1.0)	−4.2 (−5.8–−2.6)	< 0.001	—
**SSI rate (OR, 95% CI)**	2.24 (0.48–10.4)	5.64 (1.48–21.5)	12.80 (3.12–52.4)	< 0.001	—
**Return to ADL at 3 mo (OR, 95% CI)**	0.72 (0.38–1.36)	0.38 (0.18–0.80)	0.18 (0.06–0.52)	< 0.001	—
B. Lumbar fusion surgery (*n* = 336; No Maln. *n* = 101, Mild *n* = 121, Mod *n* = 74, Sev *n* = 40)					
**Poor ODI at 12 mo (OR, 95% CI)**	1.98 (1.02–3.84)	4.28 (2.12–8.64)	8.84 (3.72–21.0)	< 0.001	—
**ΔODI improvement (β, 95% CI)**	−2.80 (−5.8–0.2)	−8.40 (−12.0–−4.8)	−15.60 (−21.2–−10.0)	< 0.001	—
**Length of hospital stay (β)**	−0.8 (−1.6–0.0)	−3.2 (−4.4–−2.0)	−6.8 (−9.0 to −4.6)	< 0.001	—
**Hardware failure/non-union (OR)**	1.84 (0.28–12.1)	6.48 (1.24–33.8)	14.40 (2.64–78.6)	0.002	—
**Non-routine discharge (OR)**	2.24 (0.94–5.32)	5.64 (2.44–13.0)	12.80 (4.88–33.6)	< 0.001	—
C. Cervical spine surgery (*n* = 163; No Maln. *n* = 49, Mild *n* = 59, Mod n=34, Sev *n* = 21)					
**Poor ODI at 12 mo (OR, 95% CI)**	1.48 (0.62–3.52)	2.88 (1.12–7.40)	5.64 (1.84–17.3)	0.003	—
**ΔODI improvement (β, 95% CI)**	−1.80 (−5.2–1.6)	−5.60 (−9.8–−1.4)	−10.40 (−16.8 to −4.0)	0.002	—
**New neurological deficit (OR)**	1.92 (0.28–13.1)	4.48 (0.84–23.9)	10.24 (1.84–57.2)	0.014	—
**Ambulation** **>** **2 days (OR)**	1.64 (0.68–3.96)	3.84 (1.52–9.72)	8.40 (2.68–26.4)	< 0.001	—
D. Complex/Deformity surgery (*n* = 89; No Maln. *n* = 26, Mild *n* = 32, Mod *n* = 22, Sev *n* = 9)					
**Poor ODI at 12 mo (OR, 95% CI)**	1.68 (0.48–5.88)	3.64 (0.98–13.5)	7.84 (1.48–41.5)	0.024	—
**ΔODI improvement (β, 95% CI)**	−3.20 (−8.8–2.4)	−9.80 (−16.4 to −3.2)	−18.40 (−28.4 to −8.4)	0.001	—
**LOS (β, days)**	−1.2 (−2.8–0.4)	−4.8 (−7.2 to −2.4)	−10.2 (−14.8 to −5.6)	< 0.001	—
E. Interaction testing — surgery type × malnutrition grade					
**Surgery type** **×Malnutrition on** **ΔODI (F-stat)**	*F*_(9, 882)_ = 1.84	—	—	0.058	—
**Surgery type** **×Malnutrition on LOS (F-stat)**	*F*_(9, 882)_ = 2.64	—	—	0.005	—
**Surgery type** **×Malnutrition on ODI>40 (χ**^**2**^**)**	χ^2^ = 14.28 (df = 9)	—	—	0.114	—
F. Elderly Subgroup (Age ≥65 Years, *n* = 220)					
**Poor ODI at 12 mo — Grade 1 vs. 0 (OR)**	2.18 (0.98–4.84)	—	—	—	*p* = 0.284 vs. overall
**Poor ODI at 12 mo — Grade 2 vs. 0 (OR)**	4.84 (2.12–11.0)	—	—	< 0.001	*p* = 0.342 vs. overall
**Poor ODI at 12 mo — Grade 3 vs. 0 (OR)**	9.64 (3.48–26.7)	—	—	< 0.001	*p* = 0.218 vs. overall
**Non-routine discharge — Grade** **≥2 (OR)**	5.64 (2.84–11.2)	—	—	< 0.001	*p* = 0.162 vs. overall

All subgroup models adjusted for: age, sex, BMI, ASA score, comorbidity index, and primary spinal diagnosis. OR, Odds Ratio (binary logistic); β = unstandardized coefficient (linear regression). Interaction p-values from multiplication interaction term in combined multivariable model. SSI, Surgical Site Infection; LOS, Length of Stay; ADL, Activities of Daily Living.

In lumbar fusion patients, the effect of severe malnutrition (Grade 3 vs. Grade 0) on poor ODI outcome was most pronounced, with an adjusted OR of 8.84 (95% CI: 3.72–21.0; *p* < 0.001), and ΔODI was attenuated by a mean of 15.60 points (*p* < 0.001). Hardware failure or non-union, with direct functional implications for pain and disability outcomes, was fourteen times more common in Grade 3 relative to Grade 0 fusion patients (OR: 14.40; *p* = 0.002). Lumbar decompression patients showed a similarly significant, though somewhat attenuated, gradient, with Grade 3 patients having a 6.48-fold higher adjusted odds of poor ODI at 12 months (*p* < 0.001). In cervical spine surgery, severe malnutrition was associated with a 5.64-fold higher risk of poor outcome (*p* = 0.003) and a 10.24-fold higher risk of new neurological deficit (*p* = 0.014). Complex deformity surgery patients experienced the largest absolute ΔODI attenuation in Grade 3 (−18.40 points vs. Grade 0; *p* = 0.001), consistent with the greater physiological stress and nutritional demands of multi-level reconstructive procedures.

Formal interaction testing revealed a significant interaction between surgical procedure type and malnutrition grade on length of hospital stay [*F*_(9, 882)_ = 2.64; *p* = 0.005] but not on ΔODI (*p* = 0.058) or binary poor-ODI outcome (*p* = 0.114), suggesting that while the direction and significance of the malnutrition effect was broadly consistent across procedure types, its magnitude on length of stay varied by surgical complexity. In the elderly subgroup (age ≥ 65 years; *n* = 220), the adjusted OR for poor ODI in Grade 3 vs. Grade 0 was 9.64 (95% CI: 3.48–26.7; *p* < 0.001), with no statistically significant interaction with the overall cohort estimate (*p* = 0.218), confirming that age did not modify the fundamental malnutrition-outcome relationship (Table 9).

### Cox regression: time to functional recovery

Cox proportional hazards regression was employed to model the time-to-event outcome of functional recovery, defined as achievement of the ODI MCID (≥ 12-point improvement from baseline) within the 12-month follow-up period (Table 10). The event was observed in 746 patients (82.9%), with 154 patients (17.1%) censored at last follow-up. The global Schoenfeld residuals test confirmed that the proportional hazards assumption was met (χ^2^ = 19.2; *p* = 0.268). The adjusted model demonstrated a C-index (concordance statistic) of 0.738, indicating good discriminative ability.

**Table 10:** Cox proportional hazards regression: time to functional recovery (MCID achievement in ODI ≥ 12 points) over 12 months.

Predictor variable	Unadjusted HR (95% CI)	Unadj. *p*	Adjusted HR (95% CI)	Adj. *p*	Schoenfeld *p*	Sig.
HR<1 = slower functional recovery. Reference: no malnutrition (Grade 0). Events = MCID achievement (*n* = 746, 82.9%).						
Censored: *n* = 154.						
Malnutrition grade (Reference: no malnutrition)						
**Mild malnutrition (Grade 1)**	0.82 (0.71–0.95)	0.008	0.86 (0.73–1.01)	0.066	0.428	(^*^)
**Moderate malnutrition (Grade 2)**	0.64 (0.54–0.76)	< 0.001	0.68 (0.56–0.82)	< 0.001	0.384	^***^
**Severe malnutrition (Grade 3)**	0.42 (0.33–0.53)	< 0.001	0.46 (0.35–0.60)	< 0.001	0.312	^***^
Phenotypic nutritional criteria						
**Unintentional weight loss** **>** **10%**	0.68 (0.59–0.78)	< 0.001	0.74 (0.63–0.86)	< 0.001	0.442	^***^
**Low BMI (< 18.5 kg/m** ^**2**^ **)**	0.62 (0.50–0.76)	< 0.001	0.70 (0.56–0.88)	0.002	0.398	^***^
**Low skeletal muscle mass**	0.66 (0.57–0.76)	< 0.001	0.72 (0.62–0.84)	< 0.001	0.416	^***^
Serum biomarkers						
**Albumin** **<** **3.5 g/dL**	0.58 (0.48–0.70)	< 0.001	0.64 (0.52–0.78)	< 0.001	0.362	^***^
**Prealbumin** **<** **20 mg/dL**	0.62 (0.53–0.73)	< 0.001	0.68 (0.57–0.81)	< 0.001	0.384	^***^
**PNI** **<** **40**	0.56 (0.47–0.67)	< 0.001	0.62 (0.51–0.75)	< 0.001	0.356	^***^
**GNRI** **<** **92**	0.60 (0.51–0.71)	< 0.001	0.66 (0.55–0.79)	< 0.001	0.374	^***^
Demographic factors						
**Age** **≥65 years**	0.76 (0.66–0.88)	< 0.001	0.82 (0.70–0.96)	0.014	0.512	^*^
**Female sex**	0.96 (0.84–1.10)	0.568	0.98 (0.85–1.13)	0.782	0.624	ns
**Rural residence**	0.84 (0.73–0.97)	0.018	0.88 (0.76–1.02)	0.090	0.484	(^*^)
**Current smoker**	0.80 (0.68–0.94)	0.006	0.84 (0.72–0.98)	0.024	0.448	^*^
Comorbidities						
**Diabetes mellitus**	0.78 (0.67–0.91)	0.001	0.84 (0.71–0.99)	0.038	0.462	^*^
**Depression/Anxiety disorder**	0.70 (0.59–0.83)	< 0.001	0.76 (0.64–0.90)	0.001	0.412	^***^
**ASA** **≥III**	0.64 (0.54–0.76)	< 0.001	0.70 (0.58–0.84)	< 0.001	0.368	^***^
**Cardiovascular disease**	0.80 (0.66–0.96)	0.016	0.86 (0.70–1.04)	0.118	0.502	ns
Surgical and recovery factors						
**Fusion procedure**	0.86 (0.75–0.99)	0.034	0.90 (0.78–1.04)	0.158	0.428	ns
**Any postoperative complication**	0.60 (0.51–0.71)	< 0.001	0.66 (0.55–0.79)	< 0.001	0.356	^***^
**Surgical site infection**	0.48 (0.36–0.64)	< 0.001	0.54 (0.40–0.72)	< 0.001	0.318	^***^
**Ambulation delayed (>** **2 days)**	0.56 (0.47–0.67)	< 0.001	0.62 (0.52–0.74)	< 0.001	0.342	^***^
**Nutritional supplementation received**	1.36 (1.16–1.58)	< 0.001	1.28 (1.08–1.52)	0.004	0.578	^**^
Median time to MCID (months, 95% CI)						
**No malnutrition (Grade 0)**	3.2 (2.8–3.6)	—	—	—	—	—
**Mild malnutrition (Grade 1)**	4.8 (4.2–5.4)	—	—	—	—	—
**Moderate malnutrition (Grade 2)**	7.4 (6.6–8.2)	—	—	—	—	—
**Severe malnutrition (Grade 3)**	10.8 (9.4–12.0+)	—	—	—	—	—
**Log-rank test (overall)**	χ^2^ = 86.4	< 0.001	—	—	—	—
Model diagnostics						
**C-index (concordance statistic)**	0.694	—	0.738	—	—	—
**Global Schoenfeld test (PH assumption)**	—	—	χ^2^=19.2, p=0.268	—	—	—

Event = achievement of MCID in ODI (≥12-point improvement from baseline). Patients not achieving MCID within 12 months censored at last follow-up. Adjusted model: age, sex, BMI, diabetes, depression, ASA, surgical type, complications, grade. Schoenfeld residuals: p > 0.05 confirms proportional hazards assumption met. Significance: ^***^p < 0.001, ^**^p < 0.01, ^*^p < 0.05, (^*^) p < 0.10, ns p ≥ 0.10.

Malnutrition grade was independently associated with significantly delayed functional recovery in a dose-dependent manner. Relative to Grade 0 patients, the adjusted hazard ratios for time to MCID achievement were 0.86 (95% CI: 0.73–1.01; *p* = 0.066) for Grade 1, 0.68 (95% CI: 0.56–0.82; *p* < 0.001) for Grade 2, and 0.46 (95% CI: 0.35–0.60; *p* < 0.001) for Grade 3. The adjusted HR of 0.46 for severe malnutrition indicates that Grade 3 patients were recovering at less than half the rate of Grade 0 patients throughout the observation period. This temporal disadvantage was reflected in median times to MCID achievement of 3.2 months (Grade 0), 4.8 months (Grade 1), 7.4 months (Grade 2), and 10.8 months (Grade 3; log-rank *p* < 0.001; Table 10).

Additional independent predictors of delayed recovery in the adjusted model included SSI (HR: 0.54; *p* < 0.001), delayed ambulation (HR: 0.62 per day; *p* < 0.001), postoperative complication (HR: 0.66; *p* < 0.001), depression/anxiety (HR: 0.76; *p* = 0.001), and ASA ≥ III (HR: 0.70; *p* < 0.001). Conversely, perioperative nutritional supplementation was the only modifiable factor associated with accelerated recovery (adjusted HR: 1.28; 95% CI: 1.08–1.52; *p* = 0.004), providing time-to-event evidence that nutritional intervention shortens the recovery trajectory (Table 10).

### Mediation analysis: mechanistic pathways

Causal mediation analysis using the Baron and Kenny framework with 5,000 bootstrapped iterations was performed to identify the mechanistic pathways through which preoperative malnutrition exerts its effect on postoperative functional outcomes (Table 11). The total effect of severe malnutrition (Grade 3 vs. Grade 0) on ΔODI was −14.00 points (*p* < 0.001). Of this total effect, 58.0% was transmitted through identifiable postoperative intermediary pathways, with a total indirect effect of −8.12 points (bootstrap 95% CI: −10.64 to −5.72; *p* < 0.001). The remaining 42.0% represented the direct effect of malnutrition independent of the measured mediators.

**Table 11:** Mediation analysis: pathways through which malnutrition grade affects postoperative functional outcomes in spinal surgery patients.

Exposure → Mediator → Outcome	Total Effect (c)	Direct Effect (c')	Indirect Effect (a × b)	% Mediated	Bootstrap 95% CI	*p*-value
Outcome: ΔODI (continuous). Exposure: Malnutrition Grade (0→3 ordinal). Bootstrap iterations = 5,000. All models adjusted for						
age, sex, BMI, ASA, surgical type.						
Primary outcome: ΔODI at 12 months | Exposure: severe vs. no malnutrition						
**Pathway via postoperative complications**	−14.00	−9.52	−4.48	32.0%	−6.82 to −2.36	< 0.001
**Pathway via length of hospital stay**	−14.00	−10.08	−3.92	28.0%	−5.96 to −2.04	< 0.001
**Pathway via time to first ambulation**	−14.00	−10.64	−3.36	24.0%	−5.12 to −1.76	< 0.001
**Pathway via surgical site infection**	−14.00	−12.04	−1.96	14.0%	−3.28 to −0.76	0.002
**Pathway via postoperative delirium**	−14.00	−12.60	−1.40	10.0%	−2.48 to −0.44	0.005
**Pathway via perioperative anemia**	−14.00	−12.88	−1.12	8.0%	−2.00 to −0.32	0.008
**Total indirect effect (all mediators)**	−14.00	−5.88	−8.12	58.0%	−10.64 to −5.72	< 0.001
Primary outcome: ΔODI at 12 months | Exposure: moderate vs. no malnutrition						
**Pathway via Postoperative Complications**	−7.60	−5.22	−2.38	31.3%	−3.76 to −1.12	< 0.001
**Pathway via Length of Hospital Stay**	−7.60	−5.54	−2.06	27.1%	−3.24 to −1.02	< 0.001
**Pathway via Time to First Ambulation**	−7.60	−5.86	−1.74	22.9%	−2.74 to −0.84	0.001
**Total indirect effect (all mediators)**	−7.60	−3.44	−4.16	54.7%	−5.62 to −2.82	< 0.001
Primary Outcome: ΔODI at 12 Months | Exposure: Mild vs. no malnutrition						
**Pathway via postoperative complications**	−2.40	−1.68	−0.72	30.0%	−1.44 to −0.12	0.022
**Pathway via length of hospital stay**	−2.40	−1.74	−0.66	27.5%	−1.32 to −0.08	0.031
**Total indirect effect (all mediators)**	−2.40	−1.10	−1.30	54.2%	−2.24 to −0.44	0.004
Secondary outcome: poor ODI (binary) | Log-odds scale						
Severe Maln. ** → SSI** ** → Poor ODI**	1.732	1.302	0.430	24.8%	0.146 to 0.802	0.004
**Severe Maln**. ** → Non-routine discharge** ** → Poor ODI**	1.732	1.188	0.544	31.4%	0.224 to 0.932	0.001
**Severe Maln**. ** → Delirium** ** → Poor ODI**	1.732	1.412	0.320	18.5%	0.088 to 0.624	0.012
Secondary Outcome: EQ-5D at 12 Months | Exposure: severe vs. no malnutrition						
**Pathway via complications**	−0.312	−0.208	−0.104	33.3%	−0.162 to −0.052	< 0.001
**Pathway via ambulation delay**	−0.312	−0.228	−0.084	26.9%	−0.132 to −0.040	0.002
**Pathway via FIM (functional independence)**	−0.312	−0.248	−0.064	20.5%	−0.106 to −0.026	0.006
**Total indirect (EQ-5D)**	−0.312	−0.128	−0.184	59.0%	−0.248 to −0.124	< 0.001
Sensitivity Analyses						
Sobel test: Severe maln. ** → Complications** ** → ΔODI**	z = −5.24	—	—	—	—	< 0.001
**Sobel test: Severe maln**. ** → LOS** ** → ΔODI**	z = −4.86	—	—	—	—	< 0.001
**E-value (unmeasured confounding**, **ΔODI)**	E-value = 3.14	—	Robust to moderate confounding	—	—	—
Technical notes						
**Missing data approach**	Multiple imputation m = 20	—	Rubin's rules	—	—	—
**Bootstrapping**	5,000 iterations	—	BCa CI	—	—	—
**Software/Package**	R 4.3.1	—	mediation v4.5.0	—	—	—

Mediation analysis: Baron & Kenny framework with bootstrapped BCa confidence intervals. Positive indirect effect = mediator transmits harmful effect of malnutrition. % Mediated = indirect/(total) × 100. E-value: minimum strength of unmeasured confounding association (on RR scale) needed to explain away the indirect effect; E-value 3.14 indicates robust findings. BCa, Bias-Corrected and Accelerated bootstrap.

The single largest mediating pathway was postoperative complications (indirect effect: −4.48 points; 32.0% mediated; bootstrap 95% CI: −6.82 to −2.36; *p* < 0.001), followed by prolonged length of hospital stay (−3.92 points; 28.0%), delayed first ambulation (−3.36 points; 24.0%), surgical site infection (−1.96 points; 14.0%), postoperative delirium (−1.40 points; 10.0%), and perioperative anemia (−1.12 points; 8.0%). Sobel test validation confirmed the statistical robustness of each pathway (all z-statistics ≥ 3.94; *p* < 0.001). Comparable mediation patterns were identified for moderate malnutrition (Grade 2), where 54.7% of the total ODI effect was mediated (indirect effect: −4.16 points; *p* < 0.001), and for mild malnutrition (Grade 1), where 54.2% was mediated (indirect effect: −1.30 points; *p* = 0.004; Table 11).

Mediation analysis for the EQ-5D utility outcome revealed that postoperative complications, delayed ambulation, and impaired functional independence (FIM) together transmitted 59.0% of the total effect of severe malnutrition on quality of life at 12 months (total indirect: −0.184; *p* < 0.001). The E-value of 3.14 for the primary ODI mediation pathway indicates that an unmeasured confounder would need to be associated with both malnutrition and ΔODI by a relative risk of at least 3.14 on both pathways to fully explain the observed indirect effect a threshold considered implausible given comprehensive covariate adjustment (Table 11).

### Perioperative nutritional intervention: propensity score–matched analysis

To evaluate the real-world impact of structured perioperative nutritional support, a propensity score–matched analysis was conducted among the 630 malnourished patients (Grade ≥ 1), of whom 315 received a protocolised nutritional intervention and 315 received standard institutional care (Table 12). Matching was performed on a 1:1 nearest-neighbor basis (caliper = 0.02 SD) across age, sex, BMI, grade, primary spinal diagnosis, ASA score, surgical procedure type, diabetes status, and depression status. Post-matching balance was confirmed with all standardized mean differences (SMD) below 0.10 across all covariates, indicating adequate group comparability.

**Table 12:** Perioperative nutritional intervention impact on functional outcomes: propensity score–matched analysis among malnourished patients (Grade ≥ 1, *n* = 630).

Outcome Parameter	Nutritional Intervention (*n* = 315)	Standard Care (*n* = 315)	OR or β (95% CI)	*p*-value	NNT/ NNH
Propensity score 1:1 matched on: age, sex, BMI, grade, ASA, diagnosis, surgical type. SMD<**0.10 for all covariates post-matching**.					
Nutritional status restoration by day 7					
Albumin **≥3.5 g/dL achieved**, ***n*** **(%)**	218 (69.2%)	84 (26.7%)	OR: 5.88 (4.08–8.48)	< 0.001	NNT: 2.4
**Prealbumin** **≥20 mg/dL achieved**, ***n*** **(%)**	196 (62.2%)	68 (21.6%)	OR: 5.72 (3.92–8.36)	< 0.001	NNT: 2.5
**PNI** **≥40 achieved at discharge**, ***n*** **(%)**	228 (72.4%)	88 (27.9%)	OR: 6.92 (4.74–10.1)	< 0.001	NNT: 2.3
**Weight maintained or gained (±2%)**, ***n*** **(%)**	236 (74.9%)	148 (47.0%)	OR: 3.38 (2.38–4.80)	< 0.001	NNT: 3.6
Primary functional outcomes at 12 months					
ODI score at 12 months, mean **±SD**	27.4 ± 11.2	36.8 ± 13.8	β: −9.40 (−11.8 to −7.0)	< 0.001	—
**ΔODI improvement (pre → 12 mo), mean** **±SD**	−32.8 ± 9.8	−25.4 ± 11.4	β: +7.40 (5.0–9.8)	< 0.001	—
**MCID achievement in ODI at 12 mo**, ***n*** **(%)**	268 (85.1%)	208 (66.0%)	OR: 2.92 (2.00–4.26)	< 0.001	NNT: 5.3
**EQ-5D utility at 12 months, mean** **±SD**	0.66 ± 0.13	0.54 ± 0.15	β: +0.12 (0.09–0.15)	< 0.001	—
**VAS Back Pain at 12 months, mean** **±SD**	2.6 ± 1.3	3.8 ± 1.6	β: −1.20 (−1.58 to −0.82)	< 0.001	—
**Return to independent ADL at 3 months**, ***n*** **(%)**	248 (78.7%)	178 (56.5%)	OR: 2.86 (2.00–4.10)	< 0.001	NNT: 4.5
Complication rates (Intervention vs. control)					
Any postoperative complication, ***n*** **(%)**	68 (21.6%)	122 (38.7%)	OR: 0.44 (0.31–0.63)	< 0.001	NNT: 5.9
**Superficial or deep SSI**, ***n*** **(%)**	24 (7.6%)	62 (19.7%)	OR: 0.34 (0.20–0.56)	< 0.001	NNT: 8.3
**Wound dehiscence**, ***n*** **(%)**	8 (2.5%)	28 (8.9%)	OR: 0.26 (0.12–0.58)	0.001	NNT: 15.6
**Length of hospital stay, days, mean** **±SD**	6.8 ± 2.8	10.2 ± 4.6	β: −3.40 (−4.16 to −2.64)	< 0.001	—
**Time to first ambulation, days, mean** **±SD**	1.8 ± 0.7	2.8 ± 1.1	β: −1.00 (−1.24 to −0.76)	< 0.001	—
Healthcare Utilization					
30-day readmission, ***n*** **(%)**	24 (7.6%)	52 (16.5%)	OR: 0.42 (0.25–0.71)	0.001	NNT: 11.2
**90-day readmission**, ***n*** **(%)**	34 (10.8%)	68 (21.6%)	OR: 0.44 (0.28–0.69)	< 0.001	NNT: 9.3
**Non-routine discharge (SNF/rehab)**, ***n*** **(%)**	72 (22.9%)	148 (47.0%)	OR: 0.34 (0.24–0.48)	< 0.001	NNT: 4.2
**ICU admission required**, ***n*** **(%)**	22 (7.0%)	48 (15.2%)	OR: 0.42 (0.25–0.72)	0.001	NNT: 12.3
Grade-stratified benefit					
MCID achieved — Mild (Grade 1) intervention, ***n*** **(%)**	128 (89.5%)	108 (75.5%)	OR: 2.68 (1.44–4.98)	0.002	NNT: 7.1
**MCID achieved — Moderate (Grade 2) intervention**, ***n*** **(%)**	96 (82.1%)	72 (61.5%)	OR: 2.92 (1.68–5.08)	< 0.001	NNT: 4.9
**MCID achieved — Severe (Grade 3) intervention**, ***n*** **(%)**	44 (68.8%)	28 (43.8%)	OR: 2.84 (1.36–5.94)	0.005	NNT: 4.0
Intervention protocol details					
**ONS: high-protein supplement (≥18g protein/serving, 2 × /day)**	Delivered	—	—	—	—
**Duration: peri-operative**	7 days pre-op + 14 days post-op	—	—	—	—
**Dietitian-led counseling sessions**	Day 0, 3, 7, discharge, 4 wks	—	—	—	—
**Protein target** **≥1.5 g/kg/day achieved**, ***n*** **(%)**	278 (88.3%)	104 (33.0%)	OR: 15.8 (9.84–25.4)	< 0.001	NNT: 1.8
**Caloric target** **≥25 kcal/kg/day achieved**, ***n*** **(%)**	262 (83.2%)	128 (40.6%)	OR: 7.28 (4.92–10.8)	< 0.001	NNT: 2.4

Propensity score 1:1 nearest-neighbor matching (caliper = 0.02 SD) on: age, sex, BMI, grade, primary diagnosis, ASA score, surgical type, diabetes, depression. Post-match balance: all standardized mean differences (SMD) < 0.10. NNT = Number Needed to Treat (favorable outcome) or NNH (harmful). OR = Odds Ratio; β = mean difference (linear regression). ONS, Oral Nutritional Supplement; SSI, Surgical Site Infection; SNF, Skilled Nursing Facility.

The nutritional intervention protocol comprised high-protein oral nutritional supplementation (ONS; ≥ 18 g protein per serving, administered twice daily) combined with dietitian-led individualized counseling, initiated 7 days preoperatively and continued for 14 days postoperatively, with structured dietitian assessments at Day 0, Day 3, Day 7, and at hospital discharge. A four-week post-discharge follow-up session was also included. Protein targets of ≥ 1.5 g/kg/day were achieved in 88.3% of the intervention group compared with only 33.0% of controls (OR: 15.8; *p* < 0.001), confirming high protocol fidelity in the intervention arm (Table 12).

Nutritional status restoration was significantly more frequent in the intervention group. Albumin normalization (≥ 3.5 g/dL) was achieved in 69.2% of intervention vs. 26.7% of control patients by Day 7 (OR: 5.88; *p* < 0.001; NNT: 2.4). PNI ≥ 40 was attained at discharge in 72.4% vs. 27.9% (OR: 6.92; *p* < 0.001; NNT: 2.3; Table 12).

These biochemical improvements translated into substantially better functional outcomes at 12 months. Mean ODI was 27.4 ± 11.2 in the intervention group vs. 36.8 ± 13.8 in controls (β: −9.40; 95% CI: −11.8 to −7.0; *p* < 0.001). The ΔODI improvement was 7.40 points greater in the intervention arm (*p* < 0.001), and MCID achievement for ODI was 85.1% vs. 66.0% (OR: 2.92; *p* < 0.001; NNT: 5.3). EQ-5D utility at 12 months was 0.66 ± 0.13 vs. 0.54 ± 0.15 (β: +0.12; *p* < 0.001), and return to independent ADLs by 3 months was 78.7% vs. 56.5% (OR: 2.86; *p* < 0.001; NNT: 4.5).

The intervention also significantly reduced the clinical complication burden. Any postoperative complication occurred in 21.6% of intervention vs. 38.7% of control patients (OR: 0.44; *p* < 0.001; NNT: 5.9), and the SSI rate was halved (7.6% vs. 19.7%; OR: 0.34; *p* < 0.001; NNT: 8.3). Length of hospital stay was shortened by a mean of 3.4 days (6.8 vs. 10.2 days; *p* < 0.001), 90-day readmission was reduced (10.8% vs. 21.6%; OR: 0.44; *p* < 0.001; NNT: 9.3), and non-routine discharge was nearly halved (22.9% vs. 47.0%; OR: 0.34; *p* < 0.001; NNT: 4.2). Stratified analysis by grade demonstrated that the intervention benefit was consistent across mild, moderate, and severe malnutrition subgroups, with MCID achievement ORs of 2.68, 2.92, and 2.84 respectively — suggesting that no subgroup was too severely depleted to derive meaningful functional benefit from nutritional optimisation (Table 12).

## Discussion

As 37% of extremely malnourished patients failed to achieve a clinically meaningful improvement in disability (ODI) and 42.6% failed to enhance their quality of life, our study shows that severe malnutrition considerably limits the long-term benefits of spine surgery. These patients continued to experience moderate-to-severe pain 12 months after surgery, demonstrating that nutritional deficiencies permanently restrict a patient's ability to recover functionally. This finding is consistent with the work of Briguglio et al. (10), who showed that systemic inflammation and preoperative protein deficiency considerably lower postoperative ODI and physical health scores in elective spine surgery. Malnutrition is known to have a detrimental impact on the ability to recover physically from surgery (11). Given the phenomenological overlap between chronic malnutrition and disability, it is plausible that some patients undergoing spine surgery may present with coexisting nutritional impairment and functional disability. The clinical and surgical significance of being malnourished prior to spine surgery has already been investigated in a number of custom retrospective cohort studies, database inquiries, or prospective research projects (12, 13). Our 12-month longitudinal data, however, expands on this baseline association by demonstrating that acute nutritional deficiency creates a strict treatment ceiling, thereby keeping more than one-third of this cohort from achieving a Minimal Clinically Important Difference (MCID).

Multivariable adjusted models revealed that Grade 3 malnutrition was the single largest independent predictor of poor functional recovery (OR: 5.64), exceeding established risk variables such as advanced age, baseline comorbidities, and surgical complexity. This data is consistent with Cho et al. (14), who found that a high-risk preoperative nutritional profile remains an independent predictor of severe unfavorable outcomes in elective spine surgery, even after controlling for age and medical frailty. Additionally, Elsamadicy et al. (15) demonstrate that, even after controlling for baseline comorbidity burdens, preoperative nutritional depletion remains a strong, independent predictor of adverse events and short-term mortality among patients undergoing geriatric spine surgery.

As shown in Table 4, severe malnutrition resulted in a 59.3% total complication rate, a 24.1% major complication rate, and a 7.4% 30-day mortality rate, resulting in deep wound infections, hardware failures, and a significant increase in both hospital stay length and overall institutional costs to $54,000. This clinical and economic burden is consistent with a separate analysis by Elsamadicy et al. (16), who found that among patients undergoing surgery for spinal metastases, preoperative malnutrition is an independent driver of adverse events and an extended hospital length of stay (aRR = 3.49). In postoperative settings, malnutrition contributes to metabolic problems. Malnutrition raises the risk of multiorgan failure and widespread infection because it is linked to extended oxidative stress, immunosuppression, and delayed wound healing. Furthermore, degeneration and muscle atrophy are caused by catabolism linked to oxidative stress and malnutrition, which most likely adds to extended mechanical ventilation. Lastly, starvation and malnutrition also have an impact on the gut, causing mucosal shrinkage and malabsorption early in the hospital stay (17).

Grade ≥ 2, prealbumin, and PNI are examples of screening techniques and biomarkers that showed comparable, solo prediction accuracy, but when combined, their diagnostic performance was much enhanced. In particular, combining serum biomarkers with clinical staging gradually increased predictive power, resulting in a comprehensive multi-marker panel AUC of 0.878 (ΔAUC = +0.080; *p* < 0.001), which demonstrated lower apparent overfitting and provided more consistent preoperative risk stratification. This result is in line with Zhou et al. (18), who showed that, in comparison to standalone evaluations (OR = 3.779 vs. 1.730), combining defined malnutrition with dynamic biochemical shifts (ΔAlb) significantly improved postoperative risk discrimination. This multi-marker synergy is consistent with Yang et al. (19), who showed that separate and PNI criteria provide contradictory results with almost no diagnostic agreement (k = 0.00). Our combined panel may help address this diagnostic gap by providing complementary information across different dimensions of preoperative nutritional status. Through the application of this paradigm to an integrated multi-marker panel in our spinal surgery cohort, we verify that the mitigation of diagnostic blind spots in high-stress surgical populations requires the anchoring of clinical criteria with objective biochemical markers.

Perioperative nutritional supplementation was independently associated with a 3.64-point greater improvement in 12-month ODI scores in whole-cohort linear regression (*p* < 0.001), suggesting that preoperative nutritional optimization may represent a potentially modifiable factor associated with postoperative functional recovery. This result is consistent with the randomized controlled trial conducted by Saleh et al. (20), which found that two weeks of nutritional supplements considerably reduced the rates of reoperation, wound healing problems, and perioperative minor complications following lumbar spine surgery.

Our propensity score-matched analysis, which showed that protocol-based optimization reduced surgical site infections by half (7.6% vs. 19.7%), saved 3.4 hospital days, and increased 12-month MCID achievement (85.1% vs. 66.0%) across all malnutrition severities, conclusively validated this therapeutic efficacy. Similarly, Rigney et al. (21) showed that a focused preoperative nutrition consultation directly reduces wound-related complications in complex spine populations, whereas better PNI scores significantly lower 90-day complication probabilities. These interventional effects strongly match with these findings. People who have diminishing but not depleted physiological reserves may be the most susceptible to the negative consequences of malnutrition because they are at a turning point between poor and hopeful prognoses, where correcting malnutrition may have the biggest impact. Poor nutrition may raise the risk of unfavorable postoperative events and complications, according to a large body of data on neurosurgery and other surgical operations (22, 23).

High protein and carbohydrate consumption and immunonutrition, or adding large amounts of different amino acids and fish oil, were suggested as stages for nutritional optimization in a review since studies have shown their positive benefits on patients (24). Therefore, fulfilling calorie needs, concentrating on protein sources, and communicating with patients and their families about barriers to reaching these goals can all be part of enhancing nutritional status. These inconsistent findings demonstrate the need for more research to create and improve preoperative patient optimization guidelines and support preoperative nutrition in order to enhance patient care and reduce healthcare utilization.

### Strengths

- Monitoring long-term, patient-centered functional recovery and MCID achievement for disability (ODI) and quality of life is the study's main strength. Since our follow-up lasts for a full year, it goes far beyond the typical 30-day acute complication window.
- To separate the independent therapeutic effect of perisurgical supplements, a propensity score-matched (PSM) analysis is used to balance baseline imbalances (age, comorbidities, and surgical complexity).
- By combining phenotypic staging with biochemical markers (prealbumin and PNI) into a single panel, synergistic multi-marker screening eliminates independent diagnostic blind spots and achieves a highly selective AUC of 0.878.

### Limitations

There are various limitations to this study.

- This study is inherently vulnerable to data collecting gaps, possible charting discrepancies, and selection biases that cannot be properly controlled as in a prospective trial because it relied on a retrospective cohort analysis of data from 2020 to 2024.
- The findings may not be applied to individuals with various regional demographics, socioeconomic backgrounds, or surgical and post-operative care protocols because the data was collected from a single institutional database.
- Tracking was observational rather than a strictly controlled, blind trial, even though the analysis assessed the clinical impact of perisurgical nutritional supplements. As a result, the 12-month recovery curves may contain unmeasured confounding due to differences in actual patient compliance and dietary adherence.
- Baseline laboratory indicators obtained within a single preoperative window were used for risk classification. Rapid, acute metabolic changes or inflammatory changes that take place in the days preceding extensive spinal repair might not be adequately captured by these static baselines.
- Nutritional status, dietary intake, and supplementation were not systematically reassessed during the 12-month follow-up. Postoperative changes in these factors may have influenced functional recovery and therefore represent potential residual confounding.

## Conclusion

This study demonstrates that preoperative malnutrition, as defined by malnutrition severity criteria, acts as a strict threshold on recovery following elective spine surgery, significantly raising complications and costs while preventing more than one-third of severely depleted patients from attaining a significant improvement in disability at 12 months. This risk is, however, highly modifiable: perioperative nutritional supplementation effectively halves surgical site infections and significantly increases long-term functional recovery, while staging combined with prealbumin and PNI produces an incredibly accurate screening panel (AUC = 0.878). In order to improve patient outcomes and lessen institutional responsibilities, these data ultimately establish systematic preoperative nutritional prehabilitation as a clinical necessity. The observed associations remained directionally consistent across adjusted, complete-case, multiple-imputation, and sensitivity analyses, supporting the robustness of the association between greater malnutrition severity and poorer postoperative functional outcomes.

## Data Availability

The raw data supporting the conclusions of this article will be made available by the authors, without undue reservation.
